# A comparison of metrics and performance characteristics of different search strategies for article retrieval for a systematic review of the global epidemiology of kidney and urinary diseases

**DOI:** 10.1186/s12874-018-0569-8

**Published:** 2018-10-19

**Authors:** Boris Bikbov, Norberto Perico, Giuseppe Remuzzi, on behalf of the GBD Genitourinary Diseases Expert Group, Boris Bikbov, Claudia Cella, Monica Cortinovis, William Couser, Patricia Veronica Espindola Estevez, Flavio Gaspari, Felipe Antonio Rodriguez de Leon, Catherine Michaud, Valeria Miglioli, Christopher Murray, Mohsen Nagavi, Bishnu Pahari, Norberto Perico, Esteban Porrini, Giuseppe Remuzzi, Andrea Alejandra Panozo Rivero, Bernadette Thomas, Marcello Tonelli, Karen Courville de Vaccaro, Theo Vos, Natasha Wiebe, Sara Wulf

**Affiliations:** 1 Istituto di Ricerche Farmacologiche Mario Negri IRCCS, Via G.-B. Camozzi 3 –, 24020 Bergamo, Ranica Italy; 2 Unit of Nephrology, Dialysis and Transplantation, Azienda Socio-Sanitaria Territoriale Papa Giovanni XXIII, Bergamo, Italy; 3 L. Sacco Department of Biomedical and Clinical Sciences, University of Milan, Milan, Italy

## Abstract

**Background:**

Conducting a systematic review requires a comprehensive bibliographic search. Comparing different search strategies is essential for choosing those that cover all useful data sources. Our aim was to develop search strategies for article retrieval for a systematic review of the global epidemiology of kidney and urinary diseases, and evaluate their metrics and performance characteristics that could be useful for other systematic epidemiologic reviews.

**Methods:**

We described the methodological framework and analysed approaches applied in the previously conducted systematic review intended to obtain published data for global estimates of the kidney and urinary disease burden. We used several search strategies in PubMed and EMBASE, and compared several metrics: number needed to retrieve (NNR), number of extracted data rows, number of covered countries, and when appropriate, sensitivity, specificity, precision, and accuracy.

**Results:**

The initial search obtained 29,460 records from PubMed, and 4247 from EMBASE. After the revision, the full text of 381 and 14 articles respectively was obtained for data extraction (the percentage of useful records is 1.3% for PubMed, 0.3% for EMBASE). For PubMed we developed two search strategies and compared them with a ‘gold standard’ formed by merging their results: free word search strategy (FreeWoSS) was based on the search for keywords in all fields, and subject headings based search strategy (SuHeSS) used only MeSH-mapped conditions and countries names. SuHeSS excluded almost 15% of useful articles and data rows extracted from them, but had a lower NNR of 40 and higher specificity. FreeWoSS had better sensitivity and was able to cover the vast majority of articles and extracted data rows, but had a higher NNR of 65.

**Conclusions:**

The sensitive FreeWoSS strategy provides more data for modelling, while the more specific SuHeSS strategy could be used when resources are limited. EMBASE has limited value for our systematic review.

**Electronic supplementary material:**

The online version of this article (10.1186/s12874-018-0569-8) contains supplementary material, which is available to authorized users.

## Background

The Global Burden of Disease, Injuries and Risk Factors Study (GBD) is an outstanding initiative that currently involves over 2300 collaborators from 130 countries, under the leadership of the Institute for Health Metrics and Evaluation (IHME) of the University of Washington. At the very beginning of the GBD consortium organization, in the year 2007, the IHME asked the International Society of Nephrology (ISN) to identify the team of experts that formed the Genitourinary Diseases Expert Group (GUiDEG) to conduct a systematic review, collect data and provide their expertise regarding several conditions (see Additional file 1 Section I), including chronic kidney disease (CKD).

The results of this work, conducted jointly by GUiDEG and IHME, were used to produce estimates for the already published GBD results – covering a total of 291 diseases and injuries on a global, regional and national level for 187 countries, [1–3] – and for subsequent GBD revisions. Here we describe the methodology used in our systematic literature review on the epidemiology of kidney and urinary diseases, and provide insights into the general framework and different search strategies underlying GBD estimates that could be useful for other systematic epidemiologic reviews.

## Methods

### General framework of the systematic review

**Fig. 1:**
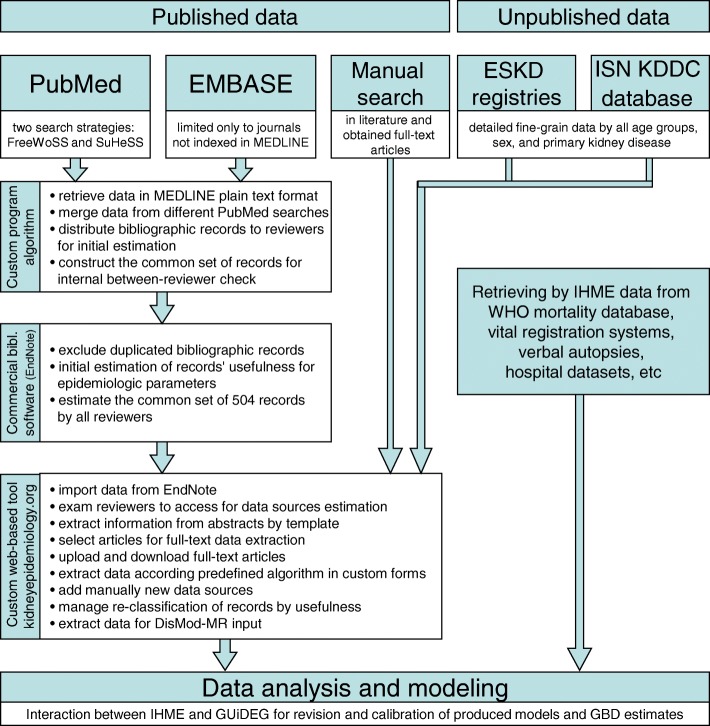
The general workflow of the GBD Study systematic review for kidney and urinary disease. ESKD – end-stage kidney disease, GUiDEG – GBD Genitourinary Diseases Expert Group, IHME – Institute for Health Metrics and Evaluation, KDDC – Kidney Disease Data Center

This systematic review consisted of several steps, with the main goal being to collect published and unpublished information about the kidney and urinary disease burden worldwide since 1980 (Fig. 1). Most of the unpublished information was collected by IHME, and we describe this outstanding work only briefly, since it requires established connections to international organizations (WHO, etc.) and agreements with governments or hospital networks. This article focuses mainly on the collection of published epidemiologic data, both the general framework and developed search strategies of which could be used by the wider scientific community.

In brief, we developed several search strategies for MEDLINE (via PubMed interface) and EMBASE, and retrieved all records in a bibliographic software (EndNote, Thomson Reuters) where we excluded duplicated records, estimated the potential usefulness of all records, and controlled the between-reviewers agreements in this step. Further, we developed a custom web-based tool (accessible via http://kidneyepidemiology.org/gbd/) that provides the opportunity for a geographically distributed team to work on the systematic review together. Using this tool, we further classified the retrieved bibliographic records, selected literature sources, managed full-text articles, and extracted epidemiologic data according to predefined templates. We also manually scanned reference lists of the obtained full-text articles, and included from there additional relevant publications for data extraction. Parallel with the data extraction from the published literature, unpublished data were obtained from all possible sources (Fig. 1). IHME obtained unpublished data from the WHO mortality database, vital registration systems, verbal autopsies and hospital datasets, and GUiDEG requested unpublished data from several end-stage kidney disease (ESKD) registries, extracted data from ESKD registry reports not indexed in the bibliographic databases, and performed the extraction of data concerning CKD prevalence in low- and middle-income countries from the Kidney Disease Data Center [4] (supported by the ISN) managed by the Mario Negri Institute for Pharmacological Research (Bergamo, Italy).

Finally, all extracted epidemiologic data were exported according to a predefined format for analysis in the DisMod meta-regression tool created by IHME, where a set of iterative models were constructed and evaluated jointly with GUiDEG. The best performance model was used to produce the final estimates on the burden of kidney and urinary diseases [1–3]. Preparation to conduct this systematic review began in September 2007, definitive bibliographic search strategies were developed and applied in April 2009, and all extracted data were submitted for DisMod-MR analysis in June 2011. The study complies with the Guidelines for Accurate and Transparent Health Estimates Reporting (GATHER) recommendations [5] (see Additional file 1 Section V), but was not registered with PROSPERO since the bibliographic search strategies were completed before it became available. Below we describe all of the steps in more detail, and present the results of this work.

#### Step 1: Developing search strategies

Our aim was to develop a highly sensitive search strategy to identify the vast majority of literature sources on the epidemiology of kidney and urinary diseases published between 1980 and 2009 without any language restrictions.

For PubMed we developed two search strategies (see Additional file 1 Section II): the free word search strategy (FreeWoSS) and the subject headings based search strategy (SuHeSS). FreeWoSS was based on the search for keywords in all fields according to the Automatic Term Mapping strategy in PubMed, including the title, abstract, and medical subject headings (MeSH) terms. This makes FreeWoSS the most sensitive and least specific strategy, which focuses on obtaining as many relevant records as possible, with the disadvantage that it retrieves many non-relevant articles. Although we used the PubMed field descriptors to restrict the search results to humans and to exclude non-relevant publication types (such as case reports, randomized controlled trials, etc.), the initial search retrieved a substantial number of clearly non-relevant data sources. A manual review of the first 1000 obtained records for each condition assigned to our group (see Additional file 1 Section I) revealed some typical keywords that were used as stop-word exclusion criteria (see Additional file 1 Section II).

SuHeSS was developed by using only MeSH-mapped conditions with the names of countries or world regions (see Additional file 1 Section II). It was expected that SuHeSS would substantially reduce the number of records retrieved from PubMed based on the filtering of literature records by the National Library of Medicine librarians, who already indexed the papers and assigned appropriate MeSH terms considering the epidemiology of the relevant conditions.

After the formulation of these two search strategies, and after creating appropriate PubMed queries, we performed a trial search using both FreeWoSS and SuHeSS for ‘Chronic kidney disease’, and evaluated the first 1000 records obtained according to predefined criteria regarding potential usefulness in order to choose a single strategy for all our conditions of interest. We found that each strategy alone (see Additional file 1 Section III) excluded a rather substantial number of potentially useful literature data sources that would prevent us from obtaining information regarding global evidence of disease burden, especially for countries or populations with a paucity of published results on the epidemiology of kidney and urinary diseases. Due to this, we decided to apply, for all our conditions, both search strategies to obtain the most comprehensive set of bibliographic records. This combined approach also provided us with the opportunity to compare different search strategies on the full set of data, which could be of interest for systematic literature reviews in general.

For EMBASE, a single search strategy was developed, similar to SuHeSS, with the exclusion of journals indexed in PubMed (see Additional file 1 Section II).

#### Step 2: Implementing search strategies

Given the substantial number of retrieved data sources in our PubMed searches we developed a special program algorithm. First, the algorithm performed a series of exchanges with the http://eutils.ncbi.nlm.nih.gov NLM server with open loop automation, passing to the NLM server the information mimicking the user input and checking the consistency of obtained results, similar to how it is described by the Hedges team [6]. Bibliographic data were retrieved from PubMed using the algorithm in the MEDLINE plain text format, and from EMBASE by the web-interface in RIS format. Finally, all search results were imported into commercial bibliographic software (EndNote), with additional mapping for the original search strategy used to obtain each record, allocation to reviewer, and selection of a common set of 504 records for estimation by all reviewers at step 3.

#### Step 3: Selection of potentially useful abstracts

Formal instructions (see Additional file 1 Section III) were developed for judging all bibliographic records based on the title and abstract, and all records were classified into a binary relevance scheme, as ‘potentially useful’ or ‘not useful’ for further consideration. At this stage, each of the four reviewers was provided with about 8300 records to classify. A common set of 504 records was provided to each reviewer to test the consistency of estimation between them. Where there were discrepancies between reviewers for these 504 records, the final decision on the allocation of the record to ‘potentially useful’ or ‘not useful’ was made during a meeting with all reviewers. The high rate of agreement between reviewers and, most importantly, high specificity and sensitivity (see Additional file 1: Table S1) confirmed that this strategy guarantees the inclusion of the greatest number of potentially useful data sources for further evaluation.

#### Step 4: Classification of potentially useful abstracts

**Fig. 2:**
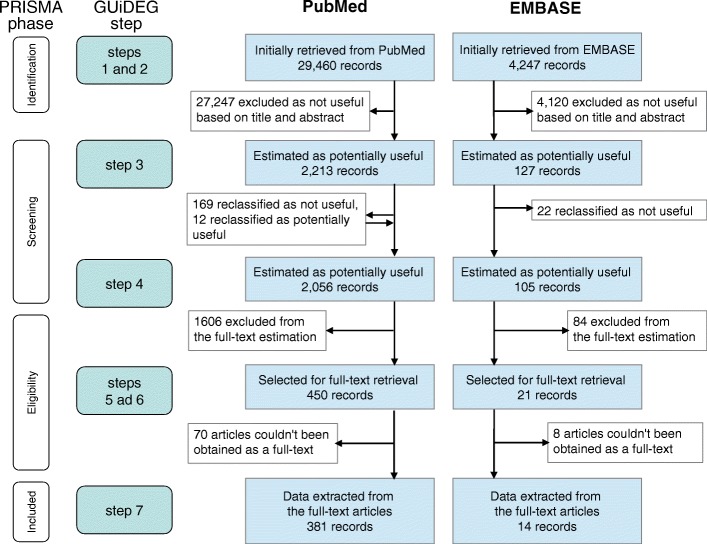
PRISMA flowchart chart for PubMed and EMBASE records in the systematic review. GUiDEG – GBD Genitourinary Diseases Expert Group

All potentially useful abstracts were further classified using a predefined strategy according to the country of description, year the study was conducted, epidemiologic parameters of interest, and other features of the studied population that could be relevant to our systematic review (see Additional file 1 Section III). This classification was performed in the web-based system (http://kidneyepidemiology.org/gbd), which provides flexibility of work distribution between reviewers, contains an online help system with examples of estimations and additional explanations for users, and has a set of tools for monitoring progress in article evaluation. Moreover, since some reviewers were unfamiliar with the methodological approach of systematic reviews and the classification of records according to developed criteria, the web-system had an obligatory examination of classification skills built in for each potential reviewer. During each examination, a potential reviewer classified 10 records that were compared with the classification performed by the experienced reviewer, and only users who passed this test were admitted to work on the real record classification. During classification, at this step it was also possible to change the allocation of any bibliographic record to ‘potentially useful’ or ‘not useful’ through a joint decision made by two reviewers in case of any previous misclassification. Due to this, 12 records were reclassified as ‘potentially useful’, while 191 were marked as ‘not useful’ for future analyses (Fig. 2).

#### Step 5: Selection of articles for the full-text retrieval

We expected a substantial proportion of potentially useful records to be present for the same study, or that there would be several bibliographic records for the same country and similar time periods but with different methodological qualities or that they would diverge substantially in terms of the size of the studied population. For this reason we implemented in the web-system a function to select only the best data sources for each country and each period of time, that lets us to avoid an excessive workload during subsequent steps in the systematic review due to the full-text extraction from several articles for the same study, or studies with inappropriate methodology, in the presence of high-quality epidemiologic studies. Based on the performed classification of abstracts, we grouped all bibliographic sources according to world region, condition of interest, country of origin, studied period, population size, and all the information available from the abstract parameters. Each potentially useful record had to be deemed as definitively useful or not useful by at least by two reviewers to be included in the full-text estimation (see Additional file 1 Section III). If two or more reviewers made a mutually exclusive decision regarding the necessity of retrieving the full text for a given literature source, the final decision was made by the third experienced reviewer. At the end of this step we formed a list of articles that had to be obtained as a full text.

#### Step 6: Retrieving full-text articles

The full-text version of papers selected in the previous step were retrieved by librarians from the Mario Negri Institute for Pharmacological Research and IHME, and were uploaded to the web-system (as PDFs, Word documents, or in scanned paper format) for further redistribution among researches involved in the full-text extraction. While we were able to obtain 395 full-text articles, there were 78 articles that couldn’t be obtained in their full-text version despite all possible efforts being made.

#### Step 7: Extraction of data from the full-text articles

The extraction was performed in the web-system (see Additional file 1 Section III) for the following epidemiologic parameters with appropriate measures of uncertainty: incidence, prevalence, mortality, patient survival, remission, cardiovascular event rate in CKD patients. Extracted data contained both general estimates for the whole studied population (for example, CKD prevalence in the whole screened cohort) and detailed estimates regarding epidemiologic parameters for subgroups defined by age, sex, race, settlement type, or geographical area described in an article. The final step was for all extracted data to be exported according IHME specifications for analysis using the DisMod meta-regression tool for producing models with epidemiologic estimates for kidney and urinary diseases. All data were controlled before inclusion in the modelling by researchers at IHME, thus providing a double check both for robustness for the full-text article selection and the extracted data.

### Performance indicators and statistical analysis for different search strategies

For each search strategy we calculated a ‘number needed to retrieve’ (NNR) metric that indicates how many records it was necessary to retrieve in the initial search to obtain one useful published article for full-text evaluation and data extraction. We prefer to use the term ‘number needed to retrieve’ instead of ‘number needed to read’ used by other authors, since the initial selection process is not related to reading full-text articles.

$$ \mathrm{Number}\ \mathrm{Needed}\ \mathrm{to}\ \mathrm{Retrieve}=\frac{\mathrm{Number}\ \mathrm{of}\ \mathrm{full}\hbox{-} \mathrm{text}\ \mathrm{articles}\ \mathrm{used}\ \mathrm{for}\ \mathrm{data}\ \mathrm{extraction}}{\mathrm{Total}\ \mathrm{number}\ \mathrm{of}\ \mathrm{records}\ \mathrm{retrieved}\ \mathrm{using}\ \mathrm{search}\ \mathrm{strategy}} $$

For the PubMed search only, we were able to form a total set by merging FreeWoSS and SuHeSS results, which we considered a ‘gold standard’ set. Using this set, we calculated sensitivity, specificity, precision, and accuracy (see Additional file 1 Section IV), as well as percentage of full-text articles and percentage of data rows excluded by each of the search strategies applied in PubMed.

A calculation of 95% CI was performed in R (v. 3.2.3) using the epiR package for sensitivity, specificity, precision and accuracy, and using the Score method [7] for percentage of useful records and NNR.

## Results

### General results

**Fig. 3:**
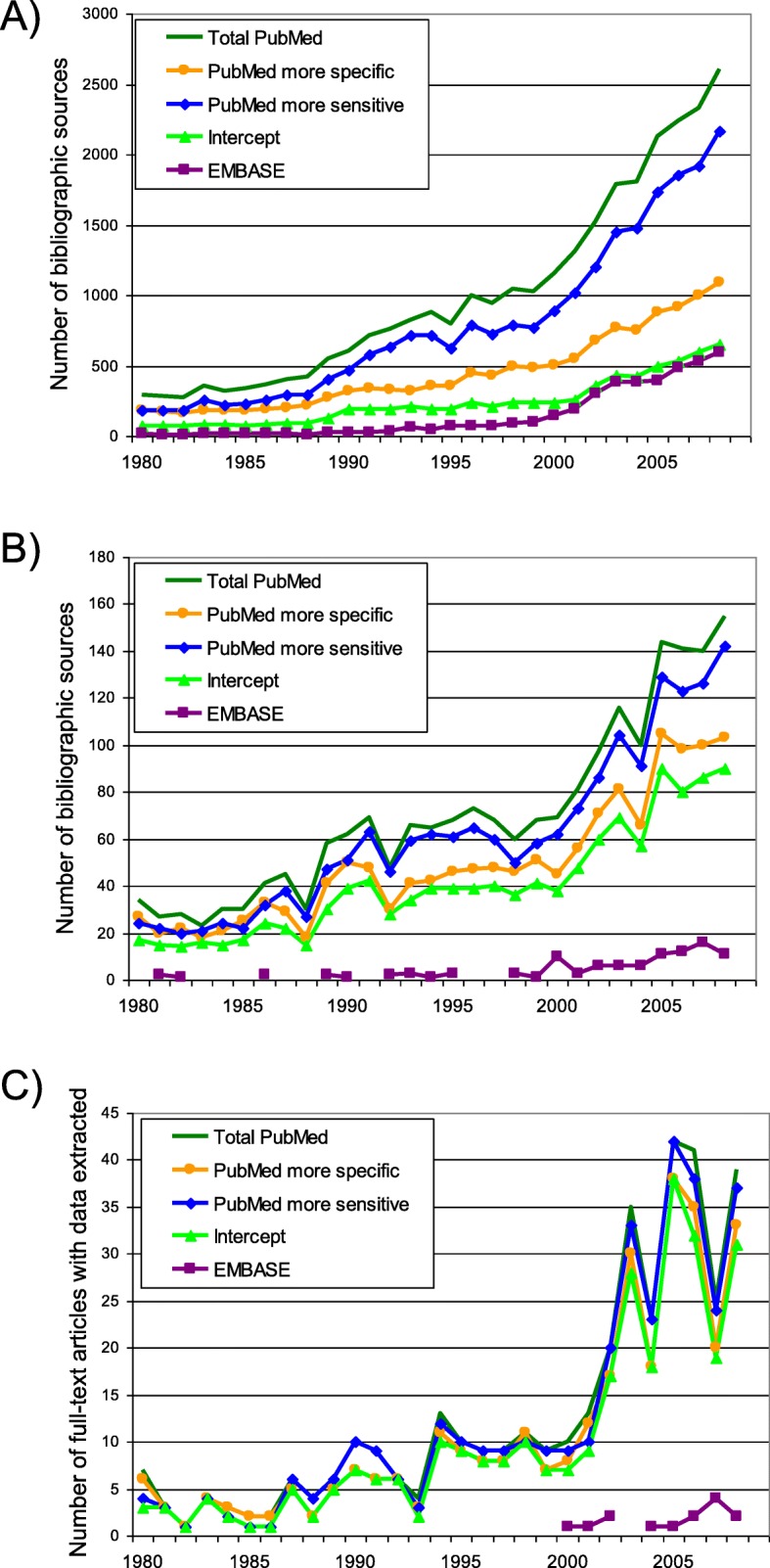
Number of published articles by search strategy and year of publication. **a** Number of records initially retrieved by search (step 2); **b** Number of records classified as potentially useful (step 3); **c** Number of useful full-text articles with data extracted (step 7). Only completely covered by the search strategies years are shown

The initial bibliographic search obtained 29,460 records from PubMed, and 4247 from EMBASE (Fig. 2). The retrieved records were published in 3447 journals, with wider coverage by PubMed (Table 1). We found a positive temporary trend in the number of articles published each year (Fig. 3a).

**Table 1:** Number of records and indicators of bibliographic search strategies applied in PubMed and EMBASE

	PubMed				EMBASE^b^	Manual search and unpublished data
	Total by merging SuHeSS and FreeWoSS	By SuHeSS	By FreeWoSS	Intercept of FreeWoSS and SuHeSS^a^		
Indicators of bibliographic search obtained by search strategy						
Number of bibliographic records						
Initial search	29,460	13,147	23,352	7039	4247	–
Potentially useful abstracts	2056	1444	1806	1194	105	–
Selected for full-text extraction	450	384	424	358	21	28
Obtained as a full text and data extracted	381	325	360	304	14	28
% from the total number of records on given step^c^						
Initial search	87.4	39.0	69.3	20.9	12.6	–
Potentially useful abstracts	95.1	66.8	83.6	55.3	4.9	–
Selected for full-text extraction	95.5	81.5	90.0	76.0	4.5	–
Obtained as a full text and data extracted	96.5	82.3	91.1	77.0	3.5	–
Number of journals covered						
Initial search	2512	1685	2154	1134	935	6^d^
Potentially useful abstracts	473	374	426	317	79	6^d^
Selected for full-text extraction	168	154	160	146	21	6^d^
Obtained as a full text and data extracted	127	116	121	111	14	6^d^
Core indicators of the search strategy based on extraction from full-text articles						
Number of countries	117	115	116	114	12	54
Number of extracted data rows	7729	6507	7612	6390	163	12,696^e^
Number of extracted data rows per one full-text article	20.3	20.0	21.1	21.0	11.6	453.4^e^
Number needed to retrieve (NNR) to obtain^f^:						
1 potentially useful article	14 (14–15)	9 (9–10)	13 (12–14)	6 (6–6)	40 (33–49)	1
1 article intended for full-text extraction	65 (60–72)	34 (31–38)	55 (50–61)	20 (18–22)	202 (130–318)	1
1 obtained full-text article with data extracted	77 (70–85)	40 (36–45)	65 (58–72)	23 (21–26)	303 (189–603)	1

^a^intercept means the set of records captured both by SuHeSS and FreeWoSS

^b^EMBASE results exclude records indexed by MEDLINE

^c^100% refers to the number obtained by both PubMed and EMBASE

^d^concern only published data

^e^high number of extracted rows and mean number of data rows per article for unpublished data resulted from extraction of information by fine grain age and sex categories based on a request from IHME for data modelling

^f^numbers in brackets indicate 95% confidence interval

*SuHeSS* Subject headings based search strategy, *FreeWoSS* Free word search strategy

Correspondence between description in the table and step number (see Methods): Initial search – step 1 and 2; Potentially useful abstracts – step 3; Selected for full-text extraction – step 5; Obtained as a full-text and data extracted – step 6 and 7

After selecting potentially useful articles containing epidemiologic parameters of interest by reviewing titles and abstracts (step 3), the number was reduced to 2056 for PubMed and 105 for EMBASE (representing 7.0% and 2.5% of the initially retrieved records, respectively), and the number of journals also declined to 473 for PubMed and 79 for EMBASE (Table 1). Among the potentially useful publications selected in this step, there was a slowly increasing trend from 1980 to 2000 in the number of publications per year, and a much steeper increase from 2000 onwards (Fig. 3b). Further selection of the most representative publications for each country’s data sources (step 5) identified 450 publications indexed by PubMed and 21 by EMBASE to be retrieved as full-text articles for data extraction. Of these, the full text of 70 PubMed articles and 8 EMBASE articles, couldn’t be retrieved in spite of all possible efforts being made.

**Fig. 4:**
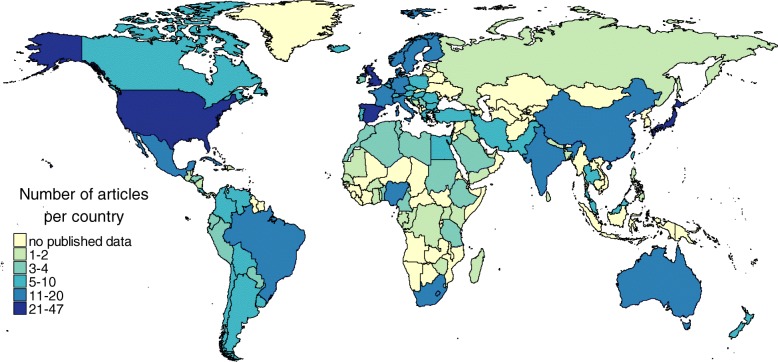
Number of articles describing each country according extraction from full-text published sources

Finally, 381 full-text articles initially found in PubMed and 14 in EMBASE were used for data extraction. Manual review of the reference list in these articles revealed another six articles that were extracted in the full-text version but were not retrieved by the initial search. Thus, the percentage of articles used for full-text extraction from the number of the records found initially were 1.3% (95%CI 1.18–1.44) for PubMed and 0.3% (95%CI 0.17–0.53) for EMBASE. The number of useful full-text papers used for data extraction increased substantially after the year 2000 (Fig. 3c). Considering the number of records in the initial search that were needed to retrieve (NRR) one article for full-text data extraction, PubMed has a much more favourable NRR of 77 (95%CI 70–85) compared with an NNR of 303 (95%CI 189–603) in EMBASE. We extracted 7729 unique data rows from articles obtained by PubMed, and only 163 data rows from those obtained by EMBASE, which corresponds to an average of 20.3 and 11.6 data rows for one full-text article, respectively. Extracted data were classified manually by country of description, with 90 and 12 countries covered in the PubMed and EMBASE sets, respectively. As many as 84 countries (43% of all countries) had no high quality published and indexed – either by PubMed or EMBASE – data on the epidemiology of kidney and urinary diseases (which especially concerns Africa and Oceania), and most data were represented by high-income countries (Fig. 4).

### Comparison of PubMed search strategies

Of the two PubMed search strategies used, the more sensitive one (FreeWoSS) retrieved 23,352 records, of which 360 (1.5%) were used for data extraction, and provided 7612 data rows for modelling. The more specific strategy (SuHeSS) retrieved 13,147 records, 325 (2.3%) of which were used for data extraction, and provided 6507 data rows. The freeWoSS and SuHeSS differed in the proportion of full-text articles used for data extraction, with 65 (95%CI 58–72) and 40 NRR (95%CI 36–45), respectively. However, the two strategies were almost completely identical in terms of the mean number of rows extracted from an article (20.0 and 21.1, respectively, *P* = 0.511), by the number of covered countries (with the exception of one country), and were very close as regards the number of journal titles covered (Table 1). Each strategy individually excluded a substantial number of full-text articles from the search results: FreeWoSS did not include 21 articles covered only by SuHeSS, while SuHeSS excluded 56 full-text articles covered only by FreeWoSS (Table 2). However, FreeWoSS did not include only 117 data rows that could be extracted only from articles obtained by SuHeSS, while SuHeSS did not include 1222 data rows that could be extracted only from articles obtained by FreeWoSS (those numbers correspond to 1.5% and 15.8% of the data rows extracted from PubMed in general, respectively).

**Table 2:** Performance of different PubMed search strategies

	SuHeSS	FreeWoSS	Intercept of FreeWoSS and SuHeSS^a^
Performance indicators (with 95% CI) of the search strategy for obtaining articles used for full-text estimation in comparison with the ‘gold standard’ set			
Sensitivity	85.3 (81.3–88.7)	94.5 (91.7–96.5)	79.8 (75.4–83.7)
Specificity	55.9 (55.3–56.5)	20.9 (20.5–21.4)	76.8 (76.3–77.3)
Precision	2.5 (2.2–2.7)	1.5 (1.4–1.7)	4.3 (3.8–4.8)
Accuracy	56.3 (55.7–56.8)	21.9 (21.4–22.4)	76.8 (76.4–77.3)
Number of bibliographic records excluded by the search strategy from the overall PubMed ‘gold standard’ set at a given step			
Initial search	16,313	6108	22,421
Potentially useful abstracts	612	250	862
Selected for full-text extraction	66	26	92
Obtained as a full-text and data extracted	56	21	77
Percentage of bibliographic records excluded by the search strategy from the overall PubMed ‘gold standard’ set at a given step			
Initial search	55.4	20.7	76.1
Potentially useful abstracts	29.8	12.2	41.9
Selected for full-text extraction	14.7	5.8	20.4
Obtained as a full text and data extracted	14.7	5.5	20.2
Extracted data rows excluded by the search strategy from the overall data rows extracted from PubMed full-text articles			
Number of data rows excluded by the strategy	1222	117	1339
Percentage of data rows excluded by the strategy	15.8	1.5	17.3

^a^intercept means the set of records captured both by SuHeSS and FreeWoSS

*SuHeSS* Subject headings based search strategy, *FreeWoSS* Free word search strategy

Correspondence between description in the table and step number (see Methods): Initial search – step 1 and 2; Potentially useful abstracts – step 3; Selected for full-text extraction – step 5; Obtained as a full-text and data extracted – step 6 and 7

We also selected the set of bibliographic records formed by the interception of FreeWoSS and SuHeSS, and this allowed us to substantially reduce the number of initial PubMed records to 7039, and keep 79.8% of all articles selected for full-text extraction (Table 1), with almost twice lower NNR of 23 compared with each PubMed search strategy per se. Intercept of search strategies contained 6390 data rows extracted from the full-text articles (82.7% of all data rows extracted from the total PubMed set), and thus excluded 1339 data rows (17.3%) that could be extracted from our whole PubMed set (Table 2).

The sensitivity of FreeWoSS was highest (Table 2), compared with it SuHeSS had lost almost 10%, and the intercept strategy almost 15% in sensitivity. The specificity was highest for the intercept strategy, almost 20% lower for SuHeSS, and 55% lower for FreeWoSS.

## Discussion

We described the general framework and details of different search strategies applied during the systematic review conducted by GUiDEG to collect evidence for the GBD study. We performed this work between 2007 and 2011, soon after the STROBE consensus guideline [8] on conducting systematic reviews of observational epidemiologic studies became available, and before the GATHER guideline was published [5]. The presented results not only satisfy the requirements of these guidelines, but also contain several innovative features. Specifically, one of our main goals was to estimate the effect of different search strategies not only on full-text article retrieval, but also regarding the number of data rows extracted from them, and their geographical coverage. For this purpose we used universal metrics to compare bibliographic search strategies: number needed to retrieve (NRR) records to obtain one full-text article for data extraction, mean number of extracted rows per article, and number of covered countries. We found that PubMed was much more efficient than EMBASE, with a NRR of 77 and 303, and a mean number of extracted rows per article of 20.3 and 11.6, respectively. To the best of our knowledge, the relative comparison of PubMed and EMBASE for searching for epidemiologic evidence has not been reported so far. The PRESS guidelines [9] recommended peer review of search strategies before conducting systematic reviews, which would improve their performance, but any data on this type of peer review is rarely mentioned in the published literature. This makes it impossible not only to judge how comprehensive a search strategy was, but also to estimate the comparative effectiveness of different systematic reviews on the same topic. Moreover, the majority of systematic reviews do not compare the effectiveness of different search strategies, and use only one without clearly defining performance indicators (such as NNR, number of extracted data rows, or number of covered countries). Nevertheless, from the articles reporting results according to PRISMA, [10] we can calculate the NNR metric for the published systematic reviews on epidemiology of certain diseases, and see substantial heterogeneity. Thus, in other systematic reviews of chronic kidney disease prevalence, the NNR varies between 42 in cases of limitation by country names [11] to 157 for a word-based strategy [12]. For the systematic reviews on acute kidney injury epidemiology, NNR varied between 12 [13] and 65 [14]. Systematic reviews performed on global epidemiology for GBD conditions by other Expert Groups had a NNR of 39 in cases of untreated caries, [15] 63 for otitis media, [16] 73 for visual impairment and blindness, [17] 132 for stroke, [18] and 220 for peripheral artery disease, [19] with wide heterogeneity in NNR also in case of risk factors presented in GBD – 29 for fasting plasma glucose and diabetes, [20] 105 for systolic blood pressure, [21] and 201 for total serum cholesterol [22]. Most systematic reviews for GBD conditions were not reported according PRISMA guidelines that do not make it possible to calculate their NNR or other bibliographic metrics.

NNR depends on many factors, including the availability of published evidence, restriction of search by controlled vocabulary of subject headings provided by the bibliographic database (MeSH in case of MEDLINE or Emtree in case of EMBASE) or other specific fields, and intercept of search terms with the common clinical terminology. Due to this, NNR could not be used to estimate the quality of a systematic review itself. Nevertheless, our analysis, which focused on systematic reviews in epidemiology, suggests that a NNR below 20 explicitly indicates the exclusion of a substantial number of useful articles, and with such a NNR, the authors of systematic reviews would need to consider making some changes to their search strategy by making it more comprehensive. Similarly, an extremely high NNR, of more than 150, would beg the question whether the search strategy was useful and cost-effective. A rather high NNR in the aforementioned systematic reviews (including ours) could be related to the frequent use of epidemiologic terminology (such as ‘incidence’, ‘prevalence’, ‘mortality’ and ‘survival’) in descriptions of clinical studies or highly selected non-representative populations that are inappropriate for epidemiologic estimates. The wide use of this terminology for clinical purposes in kidney-related literature refers to a much higher number of total records obtained in our epidemiologic search (33,707 records) compared with dentistry, [15] ophthalmology [17] or otolaryngology [16] (12,143, 14,908 and 7168 records, respectively). Restricting a search by subject headings could exclude a substantial number of useful articles and extracted from them data rows, as shown by the application of our SuHeSS strategy that did not catch almost 15% of the relevant information (Table 2). Systematic reviews in clinical fields, though to a lesser extent, also suggest the exclusion of a proportion of useful articles from the results of a search restricted to subject headings. For example, 4.2% of articles relevant to breast cancer did not have the MeSH term ‘Breast Neoplasms’, [23] and 2.6% of relevant articles on congenital vocal paralysis were not caught by the MeSH-restricted strategy [24]. Moreover, the use of MeSH precludes researchers from obtaining records that have not yet been MEDLINE-indexed, and the application of the free-text search strategy in PubMed provides, on average, an additional 160 unique records for the set of systematic reviews [25]. The negative effect of restriction by MeSH in our analysis was accompanied by the positive effect in NRR reduction to 40, implying to a reduced workload. Importantly, the intercept search strategy further reduced the NNR to 23, and excluded a percentage of relevant information similar to SuHeSS, with about 20% of useful full-text articles and data rows extracted from them compared with our ‘gold standard’ PubMed set. Thus, if there is a severe lack of resources for conducting a systematic review, or in case of a preliminary search, it is possible to suggest not using the SuHeSS strategy but applying the intercept search strategy.

The search engine interface itself could substantially influence a number of retrieved records. Because of the workload required to classification by MeSH that performed in NLM, the average time lag for a record to move from PubMed to MEDLINE In-Process was 3.3 months, and from PubMed to MEDLINE it was 10.5 months [25]. These data would favour the use of PubMed for performing systematic reviews, but a search by Ovid MEDLINE (but not Complete Ovid MEDLINE, which also covers In-Process and not indexed content) is frequently used due to its more convenient search query construction. Moreover, due to internal mechanisms, even absolutely identical queries could provide different results using different search engines, as was demonstrated by running identical searches in the Allied and Complimentary Medicine Database, which is rarely used in systematic reviews, with an almost twofold difference between records obtained by the DIALOG, Ovid and EBSCOhost interfaces [26]. The difference in the numbers of returned records between the PubMed and Ovid interfaces for the much more commonly used MEDLINE database could reach about 1% for similar strategies adapted to the interface, [27, 28] but the effect of identical search queries has not been studied yet. Excessively complex queries could substantially decrease the number of relevant articles found, and removing excessive limits by simplifying search queries could increase recall from 27 to 79% [29]. Last but not least, the availability of a uniform method of classification and terminology for describing diseases could substantially influence both the number of retrieved records and NNR, as demonstrated in our analysis by year of publication: soon after the introduction of the modern classification and the term ‘chronic kidney disease’ in 2002, it became widely used in titles, abstracts, and MeSH, which facilitated the retrieval of useful articles for data extraction. Further development of search strategies for obtaining epidemiologic evidence of disease burden would reduce the NNR to facilitate the initial steps in conducing systematic reviews, while maintaining the number of finally selected articles, data rows extracted from them, and geographical coverage.

## Conclusion

The workflow described, and the results of our search strategy, could be adapted and used for future GBD revisions, as well as being generalized for systematic literature reviews on epidemiology in other fields of medicine. We introduced new metrics to estimate the effectiveness of a given search strategy, such as number of data rows extracted from full-text articles and mean number of rows per article. These metrics, together with NNR, could be reported universally in all systematic reviews, even in the absence of a ‘gold standard’ set of records, which could facilitate comparison of different search strategies and the selection of the best strategies for future use.

## Additional file

Additional file 1: The Supplemental information contains detailed descriptions of search strategies applied to the systematic review, a description of the methodology and interface screenshots of different steps of the systematic review, the methodology used to compare different PubMed search strategies, and the Guidelines for Accurate and Transparent Health Estimates Reporting (GATHER) recommendations checklist. (PDF 1198 kb)

## Abbreviations

CKD: Chronic Kidney Disease
ESKD: End-Stage Kidney Disease
FreeWoSS: Free Word Search Strategy
GATHER: Guidelines for Accurate and Transparent Health Estimates Reporting
GBD: Global Burden of Disease, Injuries and Risk Factors Study
GUiDEG: Genitourinary Diseases Expert Group
IHME: Institute for Health Metrics and Evaluation
ISN: International Society of Nephrology
MeSH: Medical subject headings
NNR: Number Needed to Retrieve
PRISMA: Preferred Reporting Items for Systematic Reviews and Meta-Analyses
STROBE: Strengthening the Reporting of Observational Studies in Epidemiology
SuHeSS: Subject Headings Based Search Strategy
