# Supplementary material for: A comparison of metrics and performance characteristics of different search strategies for article retrieval for a systematic review of the global epidemiology of kidney and urinary diseases
Source: BMC Med Res Methodol. 2018 Oct 19;18:110. doi: 10.1186/s12874-018-0569-8 (PMC6194627; doi:10.1186/s12874-018-0569-8)
Supplement: Supplementary file 1 — The Supplemental information contains detailed descriptions of search strategies applied to the systematic review, a description of the methodology and interface screenshots of different steps of the systematic review, the methodology used to compare different PubMed search strategies, and the Guidelines for Accurate and Transparent Health Estimates Reporting (GATHER) recommendations checklist. (PDF 1198 kb) [file 12874_2018_569_MOESM1_ESM.pdf]

# Supplement

## to the article “A comparison of metrics and performance characteristics of different search strategies for article retrieval for a systematic review of the global epidemiology of kidney and urinary diseases”

## Supplement contents

I. The list of conditions assigned to the Genitourinary Diseases Expert Group systematic review

II. Search strategies applied for the systematic review

- II. 1. Free word search strategy (FreeWoSS) for PubMed
- II. 2. Subject headings search strategy (SuHeSS) for PubMed
- II. 3. Search strategy for EMBASE
- II. 4. Table with attribution of search strategies instructions to the fields of epidemiology

III. Methodology and interface of different steps of the systematic review

- III.1. Results of a trial search by both FreeWoSS and SuHeSS for the condition “Chronic kidney disease”
- III.2. Criteria for the allocation of record to “potentially useful” or “not useful” at the 3 step of the systematic review
- III.3. Results of between-reviewer agreement assessment for classification of records at the 3 step of the systematic review
- III.4. Interface for preliminary data extraction based from the abstract at the 5 step of the systematic review
- III.5. Interface for selection of articles for full-text retrieval and further data extraction at the 6 step of the systematic review
- III.6. Interface for data extraction from full-text articles at the 7 step of the systematic review

IV. Methodology for comparison of different PubMed search strategies

V. Guidelines for Accurate and Transparent Health Estimates Reporting (GATHER) recommendations checklist

## I. The list of conditions assigned to the Genitourinary Diseases Expert Group systematic review

**Box S1.** The list of conditions assigned to the Genitourinary Diseases Expert Group systematic review to collect epidemiological parameters (incidence, prevalence, mortality, survival, remission) from the full-text articles and unpublished data sources

- Chronic kidney disease (CKD) - including end-stage renal disease (ESRD), dialysis and transplantation;
- Acute glomerulonephritis;
- Chronic glomerulonephritis;
- Chronic pyelonephritis and tubulointerstitial nephritis;
- Diabetic nephropathy;
- Urolithiasis;
- Benign prostatic hypertrophy

# II. Search strategies applied for the systematic review

Below we report different search strategies, with some comments surrounded by “/* */” symbols.

## II. 1. Free word search strategy (FreeWoSS) for PubMed

```
/* Construct query for chronic kidney disease */
1. (kidney diseases) OR (chronic kidney disease) OR (chronic
kidney diseases) OR (chronic renal failure) OR (acute renal
failure) OR (acute renal failures) OR (acute kidney failure) OR
(acute kidney failures) OR (end stage renal disease) OR (end
stage renal failure) OR (end stage kidney disease) OR (end stage
kidney failure) OR (end-stage renal disease) OR (end-stage
kidney disease) OR (end-stage renal failure) OR (end-stage
kidney failure) OR "Kidney Diseases/complications"[Mesh] OR
"Kidney Diseases/epidemiology"[Mesh] OR "Kidney
Diseases/mortality"[Mesh]

/* Construct query for glomerulonephritis */
2. glomerulonephritis OR glomerulosclerosis OR (focal segmental
glomerulosclerosis) OR (lipoid nephrosis) OR (minimal change
nephropathy) OR (minimal changes disease) OR (minimal change
disease) OR (IgA nephropathy) OR (Berger Disease) OR (Bergers
Disease) OR (Berger's Disease) OR (IGA Glomerulonephritides) OR
(Immunoglobulin A Nephropathy) OR (IGA Glomerulonephritis) OR
(nephritic syndrome) OR (nephrotic syndrome) OR (glomerular
disease) OR (glomerular disorder) OR nephritis OR
"Glomerulonephritis/complications"[Mesh] OR
"Glomerulonephritis/epidemiology"[Mesh] OR
"Glomerulonephritis/mortality"[Mesh]

/* Construct query for tubulointerstitial nephritis */
3. chronic AND (pyelonephritis OR pyelitis OR (interstitial
nephritis) OR (tubulo-interstitial)) OR "Nephritis,
Interstitial/complications"[Mesh] OR "Nephritis,
Interstitial/epidemiology"[Mesh] OR "Nephritis,
Interstitial/mortality"[Mesh]

/* Construct query for urolithiasis */
4. urolithiasis OR ( (renal OR kidney OR urinary OR ureter* OR
uretra* OR bladder OR urethra) AND (calculus OR stone* OR
calculi) ) OR "Urolithiasis/complications"[Mesh] OR
"Urolithiasis/epidemiology"[Mesh]

/* Construct query for diabetic nephropathy */
5. (diabetic nephropathies) OR (diabetic kidney disease) OR
(diabetic kidney diseases) OR (diabetic nephropathy) OR
(diabetic glomerulosclerosis) OR (kimmelstiel-wilson syndrome)
OR (kimmelstiel wilson syndrome) OR (kimmelstiel-wilson disease)
OR (kimmelstiel wilson disease) OR "Diabetic
```

```
Nephropathies/complications"[Mesh] OR "Diabetic
Nephropathies/epidemiology"[Mesh] OR "Diabetic
Nephropathies/mortality"[Mesh]

/* Construct query for benign prostatic hypertrophy */
6. ((benign prostatic hypertrophy) OR (prostatic hyperplasia) OR
(prostatic hypertrophy) OR (adenofibromatous hypertrophy)) OR
"Prostatic Hyperplasia/complications"[Mesh] OR "Prostatic
Hyperplasia/epidemiology"[Mesh] OR "Prostatic
Hyperplasia/mortality"[Mesh]

/* Construct query for epidemiological parameters of interest */
7. survival OR mortality OR morbidity OR incidence OR prevalence
OR epidemiology OR screening OR (burden of disease) OR registry

/* Query for selection of registry reports */
8. (Registry OR Registries) AND (nephritis OR glomerulonephritis
OR Diabetic OR diabetes OR kidney OR pyelonephritis OR
(prostatic hypertrophy) OR urolithiasis OR dialysis OR
hemodialysis OR (kidney transplantation) OR (kidney transplant)
OR (renal failure) OR (renal insufficiency))

/* Query for restriction by year of publication */
9. "1980"[EDat] : "2010"[EDat]

/* Query for humans only */
10. "humans"[MeSH Terms]

/* Query for publication types to be excluded */
11. "Meta-Analysis"[ptyp] OR "Randomized Controlled Trial"[ptyp]
OR "Biography"[ptyp] OR "Clinical Trial, Phase I"[ptyp] OR
"Clinical Trial, Phase II"[ptyp] OR "Clinical Trial, Phase
III"[ptyp] OR "Controlled Clinical Trial"[ptyp] OR "Case
Reports"[ptyp] OR "case report"[ti]

/* Query for cancer-related issues to be excluded */
12. cancer OR neoplasm

/* Query with stop words (selected by manual classification of
first 1000 records obtained from PubMed at step 1 of systematic
review) not useful for revealing records that contain
description of epidemiological paramenters of interest */
13. (rat OR rats OR cat OR cats OR murine OR dog OR dogs OR mice
OR mouse OR salmon OR vitro) OR (transplant*[ti] NOT (kidney[ti]
OR renal[ti])) OR (calcium[ti] NOT (stone[ti] OR calcul*[ti]))
OR (phosph*[ti] NOT (stone[ti] OR calcul*[ti])) OR (contrast[ti]
AND toxicity[ti]) OR (contrast[ti] AND nephropathy[ti]) OR
(percutaneous[ti] AND coronary[ti]) OR (valve[ti] AND
surgery[ti]) OR (cardiac[ti] AND surgery[ti]) OR
(cardiothoracic[ti] AND surgery[ti]) OR (percutaneous[ti] AND
transluminal[ti] AND angioplasty[ti]) OR ("renal artery"[ti] AND
(aneurysms[ti] OR aneurysm[ti] OR restenosis[ti])) OR ((HCV[ti]
```

```
OR ("hepatitis C"[ti])) AND (dialysis[ti] OR hemodialysis[ti] OR
haemodialysis[ti] OR transplant[ti] OR transplantation[ti])) OR
((HBV[ti] OR ("hepatitis B"[ti])) AND (dialysis[ti] OR
hemodialysis[ti] OR haemodialysis[ti] OR transplant[ti] OR
transplantation[ti])) OR (genetic[ti] AND (variations[ti] OR
variation[ti])) OR (sepsis[ti] OR septic[ti] OR "Staphylococcus
aureus"[ti] OR "Escherichia coli"[ti] OR "E. coli"[ti] OR
polyomavirus[ti] OR leptospirosis[ti] OR aspergillosis[ti] OR
earthquake[ti] OR gentamycin[ti] OR cyclophosphamide[ti] OR
nephrotoxicity[ti] OR "radiocontrast-induced"[ti] OR "contrast-
induced"[ti] OR "vitamin D"[ti] OR malaria[ti] OR hemoglobin[ti]
OR haemoglobin[ti] OR anemia[ti] OR monocyte[ti] OR fetuin-A[ti]
OR 99mTc[ti] OR aneurism[ti] OR bypass[ti] OR
revascularization[ti] OR "photon emission computed
tomography"[ti] OR scintigraphy[ti] OR "aortic dissection"[ti]
OR "cardiac troponins"[ti] OR "cardiac troponin"[ti] OR
homocysteinemia[ti] OR homocystein[ti] OR osteopontin[ti] OR
osteoprotegerin[ti] OR "diabetes insipidus"[ti] OR "nephrogenic
systemic fibrosis"[ti] OR "lead intoxication"[ti] OR "post-
transplant diabetes"[ti] OR "posttransplantation"[ti] OR "dual-
kidney transplants"[ti] OR "dual-kidney transplant"[ti] OR
"pruritus"[ti] OR chromosome[ti] OR apoptosis[ti] OR
rhabdomyolysis[ti] OR "Hantaan virus"[ti] OR "Tamm-Horsfall
protein"[ti] OR "autosomal dominant polycystic kidney
disease"[ti] OR ADPKD[ti] OR ARPKD[ti] OR AIDS[ti] OR HIV[ti] OR
lupus[ti] OR SLE[ti] OR Gadolinium[ti] OR "nephrogenic systemic
fibrosis"[ti])

/* Combining queries for all conditions except prostatic
hypertrophy */
14. 1 OR 2 OR 3 OR 4 OR 5

/* Restricting to non cancer */
15. 14 NOT 12

/* Combining all conditions with prostatic hypertrophy */
16. 15 OR 6

/* Combining all conditions with registry reports */
17. 16 OR 8

/* Restricting to epidemiological parameters of interest */
18. 17 AND 7

/* Restricting to publication types */
19. 18 NOT 11

/* Restricting to stop words */
20. 19 NOT 13

/* Restricting to humans */
21. 20 AND 10
```

```
/* Restricting to date */
22. 21 AND 9
```

## II. 2. Subject headings search strategy (SuHeSS) for PubMed

```
/* Query with countries and regions names */
1. "Angola"[Mesh] OR "Afghanistan"[Mesh] OR "Africa South of the Sahara"[Mesh] OR "Africa, Central"[Mesh] OR "Africa, Eastern"[Mesh] OR "Africa, Northern"[Mesh] OR "Africa, Southern"[Mesh] OR "Africa, Western"[Mesh] OR "Africa"[Mesh] OR "Albania"[Mesh] OR "Algeria"[Mesh] OR "American Samoa"[Mesh] OR "Andorra"[Mesh] OR "Antigua and Barbuda"[Mesh] OR "Argentina"[Mesh] OR "Armenia"[Mesh] OR "Aruba"[Mesh] OR "Asia, Central"[Mesh] OR "Asia, Southeastern"[Mesh] OR "Asia, Western"[Mesh] OR "Asia"[Mesh] OR "Atlantic Islands"[Mesh] OR "Australasia"[Mesh] OR "Australia"[Mesh] OR "Austria"[Mesh] OR "Azerbaijan"[Mesh] OR "Bahamas"[Mesh] OR "Bahrain"[Mesh] OR "Baltic States"[Mesh] OR "Bangladesh"[Mesh] OR "Barbados"[Mesh] OR "Belgium"[Mesh] OR "Belize"[Mesh] OR "Benin"[Mesh] OR "Bermuda"[Mesh] OR "Bhutan"[Mesh] OR "Bolivia"[Mesh] OR "Borneo"[Mesh] OR "Botswana"[Mesh] OR "Brazil"[Mesh] OR "British Virgin Islands"[Mesh] OR "Brunei"[Mesh] OR "Bulgaria"[Mesh] OR "Burkina Faso"[Mesh] OR "Burundi"[Mesh] OR "Byelarus"[Mesh] OR "Cambodia"[Mesh] OR "Cameroon"[Mesh] OR "Canada"[Mesh] OR "Cape Verde"[Mesh] OR "Caribbean Region"[Mesh] OR "Cayman Islands"[Mesh] OR "Central African Republic"[Mesh] OR "Central America"[Mesh] OR "Chad"[Mesh] OR "Chile"[Mesh] OR "China"[Mesh] OR "Colombia"[Mesh] OR "Comoros"[Mesh] OR "Congo"[Mesh] OR "Costa Rica"[Mesh] OR "Cote d'Ivoire"[Mesh] OR "Croatia"[Mesh] OR "Cuba"[Mesh] OR "Cyprus"[Mesh] OR "Czech Republic"[Mesh] OR "Czechoslovakia"[Mesh] OR "Denmark"[Mesh] OR "Dominica"[Mesh] OR "Dominican Republic"[Mesh] OR "East Timor"[Mesh] OR "Ecuador"[Mesh] OR "Egypt"[Mesh] OR "El Salvador"[Mesh] OR "England"[Mesh] OR "Equatorial Guinea"[Mesh] OR "Eritrea"[Mesh] OR "Estonia"[Mesh] OR "Ethiopia"[Mesh] OR "Europe, Eastern"[Mesh] OR "Falkland Islands"[Mesh] OR "Far East"[Mesh] OR "Fiji"[Mesh] OR "Finland"[Mesh] OR "France"[Mesh] OR "French Guiana"[Mesh] OR "French Polynesia"[Mesh] OR "Gabon"[Mesh] OR "Gambia"[Mesh] OR "Georgia (Republic)"[Mesh] OR "Germany"[Mesh] OR "Ghana"[Mesh] OR "Gibraltar"[Mesh] OR "Great Britain"[Mesh] OR "Greece"[Mesh] OR "Greenland"[Mesh] OR "Grenada"[Mesh] OR "Guadeloupe "[Mesh] OR "Guam"[Mesh] OR "Guatemala"[Mesh] OR "Guinea"[Mesh] OR "Guyana"[Mesh] OR "Haiti"[Mesh] OR "Hawaii"[Mesh] OR "Honduras"[Mesh] OR "Hong Kong"[Mesh] OR "Hungary"[Mesh] OR "Iceland"[Mesh] OR "India"[Mesh] OR "Indian Ocean Islands"[Mesh] OR "Indonesia"[Mesh] OR "Iran"[Mesh] OR "Iraq"[Mesh] OR "Ireland"[Mesh] OR "Israel"[Mesh] OR "Italy"[Mesh] OR "Jamaica"[Mesh] OR "Japan"[Mesh] OR "Jordan"[Mesh] OR "Kazakhstan"[Mesh] OR "Kenya"[Mesh] OR "Kiribati"[Mesh] OR "Korea"[Mesh] OR "Kosovo"[Mesh] OR "Kuwait"[Mesh] OR "Kyrgyzstan"[Mesh] OR "Laos"[Mesh] OR "Latin
```

```
America"[Mesh] OR "Latvia"[Mesh] OR "Lebanon"[Mesh] OR
"Lesotho"[Mesh] OR "Liberia"[Mesh] OR "Libya"[Mesh] OR
"Liechtenstein"[Mesh] OR "Lithuania"[Mesh] OR "Luxembourg"[Mesh]
OR "Macedonia"[Mesh] OR "Madagascar"[Mesh] OR "Malawi"[Mesh] OR
"Malaysia"[Mesh] OR "Maldives"[Mesh] OR "Mali"[Mesh] OR
"Malta"[Mesh] OR "Marshall Islands"[Mesh] OR "Martinique"[Mesh]
OR "Mauritania"[Mesh] OR "Mauritius"[Mesh] OR "Mediterranean
Region"[Mesh] OR "Mexico"[Mesh] OR "Micronesia"[Mesh] OR "Middle
East"[Mesh] OR "Moldova"[Mesh] OR "Monaco"[Mesh] OR
"Mongolia"[Mesh] OR "Montenegro"[Mesh] OR "Montserrat"[Mesh] OR
"Morocco"[Mesh] OR "Mozambique"[Mesh] OR "Myanmar"[Mesh] OR
"Namibia"[Mesh] OR "Nauru"[Mesh] OR "Nepal"[Mesh] OR
"Netherlands Antilles"[Mesh] OR "Netherlands"[Mesh] OR "New
Caledonia"[Mesh] OR "New Zealand"[Mesh] OR "Nicaragua"[Mesh] OR
"Niger"[Mesh] OR "Nigeria"[Mesh] OR "North America"[Mesh] OR
"Norway"[Mesh] OR "Oman"[Mesh] OR "Pacific Islands"[Mesh] OR
"Pakistan"[Mesh] OR "Palau"[Mesh] OR "Panama"[Mesh] OR "Papua
New Guinea"[Mesh] OR "Paraguay"[Mesh] OR "Peru"[Mesh] OR
"Philippines"[Mesh] OR "Poland"[Mesh] OR "Polynesia"[Mesh] OR
"Portugal"[Mesh] OR "Puerto Rico"[Mesh] OR "Qatar"[Mesh] OR
"Reunion"[Mesh] OR "Romania"[Mesh] OR "Russia"[Mesh] OR
"Rwanda"[Mesh] OR "Saint Kitts and Nevis"[Mesh] OR "Saint
Lucia"[Mesh] OR "Saint Pierre and Miquelon"[Mesh] OR "Saint
Vincent and the Grenadines"[Mesh] OR "Samoa"[Mesh] OR "San
Marino"[Mesh] OR "Saudi Arabia"[Mesh] OR "Scandinavia"[Mesh] OR
"Senegal"[Mesh] OR "Serbia"[Mesh] OR "Seychelles"[Mesh] OR
"Sierra Leone"[Mesh] OR "Singapore"[Mesh] OR "Slovakia"[Mesh] OR
"Slovenia"[Mesh] OR "Solomon Islands"[Mesh] OR "Somalia"[Mesh]
OR "South Africa"[Mesh] OR "South America"[Mesh] OR
"Spain"[Mesh] OR "Sri Lanka"[Mesh] OR "Sudan"[Mesh] OR
"Suriname"[Mesh] OR "Swaziland"[Mesh] OR "Sweden"[Mesh] OR
"Switzerland"[Mesh] OR "Syria"[Mesh] OR "Taiwan"[Mesh] OR
"Tajikistan"[Mesh] OR "Tanzania"[Mesh] OR "Thailand"[Mesh] OR
"Timor-Leste"[Mesh] OR "Togo"[Mesh] OR "Tokelau"[Mesh] OR
"Tonga"[Mesh] OR "Trinidad and Tobago"[Mesh] OR "Tunisia"[Mesh]
OR "Turkey"[Mesh] OR "Turkmenistan"[Mesh] OR "Turks and Caicos
Islands"[Mesh] OR "Tuvalu"[Mesh] OR "Uganda"[Mesh] OR
"Ukraine"[Mesh] OR "United Arab Emirates"[Mesh] OR "United
States"[Mesh] OR "Uruguay"[Mesh] OR "USSR"[Mesh] OR
"Uzbekistan"[Mesh] OR "Vanuatu"[Mesh] OR "Vatican City "[Mesh]
OR "Venezuela"[Mesh] OR "Vietnam"[Mesh] OR "Virgin Islands of
the United States"[Mesh] OR "West Indies"[Mesh] OR "World
Health"[Mesh] OR "Yemen"[Mesh] OR "Yugoslavia"[Mesh] OR
"Zambia"[Mesh] OR "Zimbabwe"[Mesh]

/* Query for chronic kidney disease */
2. "Kidney Diseases"[Mesh]

/* Query for glomerulonephritis */
3. "Glomerulonephritis"[Mesh]

/* Query for tubulointerstitial nephritis */
```

```
4. "Pyelonephritis"[Mesh]

/* Query for urolithiasis */
5. "Urolithiasis"[Mesh]

/* Query for diabetic nephropathy */
6. "Diabetic Nephropathies"[Mesh]

/* Query for benign prostatic hypertrophy */
7. "Prostatic Hyperplasia"[Mesh]

/* Construct query for epidemiological parameters of interest */
8. survival OR mortality OR morbidity OR incidence OR prevalence
OR epidemiology OR screening OR (burden of disease) OR registry

/* Query for restriction by year of publication */
9. "1980"[EDat] : "2010"[EDat]

/* Query for humans only */
10. "humans"[MeSH Terms]

/* Query for publication types to be excluded */
11. "Meta-Analysis"[ptyp] OR "Randomized Controlled Trial"[ptyp]
OR "Biography"[ptyp] OR "Clinical Trial, Phase I"[ptyp] OR
"Clinical Trial, Phase II"[ptyp] OR "Clinical Trial, Phase
III"[ptyp] OR "Controlled Clinical Trial"[ptyp] OR "Case
Reports"[ptyp] OR "case report"[ti]

/* Query for cancer-related issues to be excluded */
12. cancer OR neoplasm

/* Query with stop words (selected by manual classification of
first 1000 records obtained from PubMed at step 1 of systematic
review) not useful for revealing records that contain
description of epidemiological paramenters of interest */
13. (rat OR rats OR cat OR cats OR murine OR dog OR dogs OR mice
OR mouse OR salmon OR vitro) OR (transplant*[ti] NOT (kidney[ti]
OR renal[ti])) OR (calcium[ti] NOT (stone[ti] OR calcul*[ti]))
OR (phosph*[ti] NOT (stone[ti] OR calcul*[ti])) OR (contrast[ti]
AND toxicity[ti]) OR (contrast[ti] AND nephropathy[ti]) OR
(percutaneous[ti] AND coronary[ti]) OR (valve[ti] AND
surgery[ti]) OR (cardiac[ti] AND surgery[ti]) OR
(cardiothoracic[ti] AND surgery[ti]) OR (percutaneous[ti] AND
transluminal[ti] AND angioplasty[ti]) OR ("renal artery"[ti] AND
(aneurysms[ti] OR aneurysm[ti] OR restenosis[ti])) OR ((HCV[ti]
OR ("hepatitis C"[ti])) AND (dialysis[ti] OR hemodialysis[ti] OR
haemodialysis[ti] OR transplant[ti] OR transplantation[ti])) OR
((HBV[ti] OR ("hepatitis B"[ti])) AND (dialysis[ti] OR
hemodialysis[ti] OR haemodialysis[ti] OR transplant[ti] OR
transplantation[ti])) OR (genetic[ti] AND (variations[ti] OR
variation[ti])) OR (sepsis[ti] OR septic[ti] OR "Staphylococcus
aureus"[ti] OR "Escherichia coli"[ti] OR "E. coli"[ti] OR
```

```
polyomavirus[ti] OR leptospirosis[ti] OR aspergillosis[ti] OR
earthquake[ti] OR gentamycin[ti] OR cyclophosphamide[ti] OR
nephrotoxicity[ti] OR "radiocontrast-induced"[ti] OR "contrast-
induced"[ti] OR "vitamin D"[ti] OR malaria[ti] OR hemoglobin[ti]
OR haemoglobin[ti] OR anemia[ti] OR monocyte[ti] OR fetuin-A[ti]
OR 99mTc[ti] OR aneurism[ti] OR bypass[ti] OR
revascularization[ti] OR "photon emission computed
tomography"[ti] OR scintigraphy[ti] OR "aortic dissection"[ti]
OR "cardiac troponins"[ti] OR "cardiac troponin"[ti] OR
homocysteinemia[ti] OR homocystein[ti] OR osteopontin[ti] OR
osteoprotegerin[ti] OR "diabetes insipidus"[ti] OR "nephrogenic
systemic fibrosis"[ti] OR "lead intoxication"[ti] OR "post-
transplant diabetes"[ti] OR "posttransplantation"[ti] OR "dual-
kidney transplants"[ti] OR "dual-kidney transplant"[ti] OR
"pruritus"[ti] OR chromosome[ti] OR apoptosis[ti] OR
rhabdomyolysis[ti] OR "Hantaan virus"[ti] OR "Tamm-Horsfall
protein"[ti] OR "autosomal dominant polycystic kidney
disease"[ti] OR ADPKD[ti] OR ARPKD[ti] OR AIDS[ti] OR HIV[ti] OR
lupus[ti] OR SLE[ti] OR Gadolinium[ti] OR "nephrogenic systemic
fibrosis"[ti])

/* Combining queries for all conditions except prostatic
hypertrophy */
14. 2 OR 3 OR 4 OR 5 OR 6

/* Restricting to non cancer */
15. 14 NOT 12

/* Combining all conditions with prostatic hypertrophy */
16. 15 OR 7

/* Restricting to countries */
17. 16 AND 1

/* Restricting to epidemiological parameters of interest */
18. 17 AND 8

/* Restricting to date */
19. 18 AND 9

/* Restricting to humans */
20. 19 AND 10

 /* Combining queries for terms to be excluded */
21. 11 OR 13

 /* Final query */
22. 20 NOT 21
```

## II. 3. Search strategy for EMBASE

```
/* Construct query for chronic kidney disease */
1. 'kidney disease'/exp

/* Construct query for glomerulonephritis */
2. ((('glomerulonephritis':ab OR 'glomerulosclerosis':ab OR
('focal segmental glomerulosclerosis':ab) OR ('lipoid
nephrosis':ab) OR ('minimal change nephropathy':ab) OR ('minimal
changes disease':ab) OR ('minimal change disease':ab) OR ('iga
nephropathy':ab) OR ('berger disease':ab) OR ('bergers
disease':ab) OR ('iga glomerulonephritides':ab) OR
('immunoglobulin a nephropathy':ab) OR ('iga
glomerulonephritis':ab) OR ('nephritic syndrome':ab) OR
('nephrotic syndrome':ab) OR ('glomerular disease':ab) OR
('glomerular disorder':ab) OR 'nephritis':ab))) OR
((('glomerulonephritis':ti OR 'glomerulosclerosis':ti OR ('focal
segmental glomerulosclerosis':ti) OR ('lipoid nephrosis':ti) OR
('minimal change nephropathy':ti) OR ('minimal changes
disease':ti) OR ('minimal change disease':ti) OR ('iga
nephropathy':ti) OR ('berger disease':ti) OR ('bergers
disease':ti) OR ('iga glomerulonephritides':ti) OR
('immunoglobulin a nephropathy':ti) OR ('iga
glomerulonephritis':ti) OR ('nephritic syndrome':ti) OR
('nephrotic syndrome':ti) OR ('glomerular disease':ti) OR
('glomerular disorder':ti) OR 'nephritis':ti))

/* Construct query for urolithiasis */
3. 'urolithiasis'/exp

/* Construct query for benign prostatic hypertrophy */
4. 'prostate hypertrophy'/exp

/* Construct query for epidemiological parameters of interest OR
registry reports */
5. ('survival'/exp OR 'mortality'/exp OR 'morbidity'/exp OR
'incidence'/exp OR 'prevalence'/exp OR 'epidemiology'/exp OR
'screening'/exp OR (burden AND of AND 'disease'/exp) OR
'registry'/exp)

/* Query for selection of registry reports */
6. (registry OR registries) AND (nephritis OR glomerulonephritis
OR diabetic OR diabetes OR kidney OR pyelonephritis OR
(prostatic AND hypertrophy) OR urolithiasis OR dialysis OR
hemodialysis OR (kidney AND transplantation) OR (kidney AND
transplant) OR (renal AND failure) OR (renal AND insufficiency))

/* Query for cancer-related issues to be excluded */
7. ('cancer':ab OR 'neoplasms':ab) OR ('cancer':ti OR
'neoplasms':ti)

/* Combining queries for all conditions except prostatic
hypertrophy */
```

```
8. 1 OR 2 OR 3

/* Restricting to non cancer */
9. 8 NOT 7

/* Combining all conditions with prostatic hypertrophy */
10. 9 OR 4

/* Combining all conditions with registries reports */
11. 10 OR 6

/* Restricting to epidemiological parameters of interest */
12. 11 AND 5

/* Restricting to year of publication */
13. 12 AND [1980-2009]/py

/* Restricting to humans only */
14. 13 AND [humans]/lim

/* Query for publication types to be excluded */
15. ([cochrane review]/lim OR [controlled clinical trial]/lim OR
[meta analysis]/lim OR [randomized controlled trial]/lim OR
[systematic review]/lim) AND [1980-2009]/py

/* Query for publication types to be excluded - 2 */
16. 'case report':ti AND [1980-2009]/py

/* Query with stop words (selected by manual classification of
first 1000 records obtained from PubMed at step 1 of systematic
review) not useful for revealing records that contain
description of epidemiological paramenters of interest */
17. ((('calcium':ti NOT ('stone':ti OR 'calculi':ti))) OR
(('phosphorus':ti NOT ('stone':ti OR 'calculi':ti))) OR
(('phosphate':ti NOT ('stone':ti OR 'calculi':ti))) OR
(('transplant':ti NOT ('kidney':ti OR 'renal':ti))) OR
(('transplants':ti NOT ('kidney':ti OR 'renal':ti))) OR
(('transplanted':ti NOT ('kidney':ti OR 'renal':ti))) OR
(('transplantation':ti NOT ('kidney':ti OR 'renal':ti))) OR
(('contrast':ti AND 'toxicity':ti) OR ('contrast':ti AND
'nephropathy':ti) OR ('percutaneous':ti AND 'coronary':ti) OR
('valve':ti AND 'surgery':ti) OR ('cardiac':ti AND 'surgery':ti)
OR ('cardiothoracic':ti AND 'surgery':ti) OR ('percutaneous':ti
AND 'transluminal':ti AND 'angioplasty':ti) OR 'renal artery':ti
AND ('aneurysms':ti OR 'aneurysm':ti OR 'restenosis':ti) OR
('hcv':ti OR ('hepatitis c':ti)) AND ('dialysis':ti OR
'hemodialysis':ti OR 'haemodialysis':ti OR 'transplant':ti OR
'transplantation':ti) OR ('hbv':ti OR ('hepatitis c':ti)) AND
('dialysis':ti OR 'hemodialysis':ti OR 'haemodialysis':ti OR
'transplant':ti OR 'transplantation':ti) OR ('genetic':ti AND
('variations':ti OR 'variation':ti))) OR ('sepsis':ti OR
'septic':ti OR 'staphylococcus aureus':ti OR 'escherichia
```

```
coli':ti OR 'e. coli':ti OR 'polyomavirus':ti OR
'leptospirosis':ti OR 'aspergillosis':ti OR 'earthquake':ti OR
'gentamycin':ti OR 'cyclophosphamide':ti OR 'nephrotoxicity':ti
OR 'radiocontrast-induced':ti OR 'contrast-induced':ti OR
'vitamin d':ti OR 'malaria':ti OR 'hemoglobin':ti OR
'haemoglobin':ti OR 'anemia':ti OR 'monocyte':ti OR 'fetuin-
a':ti OR '99mtc':ti OR 'aneurism':ti OR 'bypass':ti OR
'revascularization':ti OR 'photon emission computed
tomography':ti OR 'scintigraphy':ti OR 'aortic dissection':ti OR
'cardiac troponins':ti OR 'cardiac troponin':ti OR
'homocysteinemia':ti OR 'homocystein':ti OR 'osteopontin':ti OR
'osteoprotegerin':ti OR 'diabetes insipidus':ti OR 'nephrogenic
systemic fibrosis':ti OR 'lead intoxication':ti OR 'post-
transplant diabetes':ti OR 'posttransplantation':ti OR 'dual-
kidney transplants':ti OR 'dual-kidney transplant':ti OR
'pruritus':ti OR 'chromosome':ti OR 'apoptosis':ti OR
'rhabdomyolysis':ti OR 'hantaan virus':ti OR 'tamm-horsfall
protein':ti OR 'autosomal dominant polycystic kidney disease':ti
OR 'adpkd':ti OR 'arpkd':ti OR 'aids':ti OR 'hiv':ti OR
'lupus':ti OR 'sle':ti OR 'gadolinium':ti OR 'nephrogenic
systemic fibrosis':ti)) AND [1980-2009]/py

/* Query with stop words - 2 */
18. (('rat'/exp OR 'rat') OR ('rats'/exp OR 'rats') OR
('cat'/exp OR 'cat') OR ('cats'/exp OR 'cats') OR murine OR
('dog'/exp OR 'dog') OR ('dogs'/exp OR 'dogs') OR ('mice'/exp OR
'mice') OR ('mouse'/exp OR 'mouse') OR ('salmon'/exp OR
'salmon') OR vitro) AND [1980-2009]/py

/* Combining all exclusion criteria */
19. 15 OR 16 OR 17 OR 18

/* Restricting to stop words and exclusion conditions */
20. 14 NOT 19

/* initially for excluding from the EMBASE search all journals
that are already indexed by PubMed, we were intended to use
EMBASE instruction [embase]/lim. But manual review of obtained
articles had shown that even with this instruction applied the
EMBASE provided records from the MEDLINE-indexed journals.
Because of this, we prepared a list of journal titles indexed
only by EMBASE, based on the information available at the
official EMBASE web-site (currently this list could be found at
https://www.elsevier.com/solutions/embase-biomedical-
research/embase-coverage-and-content page, accessed 28/11/2017).
At least at the moment of search conduction, EMBASE could’t
processed all journal titles in a single query separated by
“OR”, and due to this we split journal titles for several
quesries, and later merged them. */

21. 'clinical and translational science':jt OR 'clinical
schizophrenia and related psychoses':jt OR 'cold spring harbor
```

protocols':jt OR 'current protocols in microbiology':jt OR 'current topics in neurochemistry':jt OR 'current topics in peptide and protein research':jt OR 'current topics in toxicology':jt OR 'diabetologie und stoffwechsel':jt OR 'dolor, clinica y terapia':jt OR 'drug r and d backgrounders':jt OR 'early intervention in psychiatry':jt OR 'european journal of mental health':jt OR 'folia gastroenterologica et hepatologica':jt OR 'forum kardiologow':jt OR 'gaceta mexicana de oncologia':jt OR 'genetically modified organisms':jt OR 'ginekologia i poloznictwo':jt OR 'hacettepe university journal of the faculty of pharmacy':jt OR 'hepatology reviews':jt OR 'indian journal of forensic medicine and toxicology':jt OR 'influenza and other respiratory viruses':jt OR 'international journal of mental health systems':jt OR 'international journal of probiotics and prebiotics':jt OR 'iranian journal of biotechnology':jt OR 'iranian journal of fertility and sterility':jt OR 'iranian journal of reproductive medicine':jt OR 'italian journal of medicine':jt OR 'journal of biological engineering':jt OR 'journal of biomedical nanotechnology':jt OR 'journal of biopharmaceutics and biotechnology':jt OR 'journal of cell and molecular biology':jt OR 'journal of diabetes science and technology':jt OR 'journal of medical case reports':jt OR 'journal of medical devices, transactions of the asme':jt OR 'journal of metabolism and genetics':jt OR 'journal of pure and applied microbiology':jt OR 'journal of veterinary pharmacology and toxicology':jt OR 'journal of women':jt OR 'kranion':jt OR 'maghreb medical':jt OR 'menopause review':jt OR 'mental health in family medicine':jt OR 'molecular and cellular toxicology':jt OR 'mycotoxin research':jt OR 'neurologia, neurocirugia y psiquiatria':jt OR 'neuroscience imaging':jt OR 'nonlinear biomedical physics':jt OR 'obstetrics and gynaecology forum':jt OR 'pakistan journal of biotechnology':jt OR 'pediatric rheumatology':jt OR 'pet clinics':jt OR 'pharmaceutical medicine':jt OR 'pharmacopeial forum':jt OR 'progress in neurology and psychiatry':jt OR 'przeglad kardiodiabetologinczny':jt OR 'psicooncologia':jt OR 'psychiatria':jt OR 'rehabilitation oncology':jt OR 'revista mexicana de angiologia':jt OR 'revista mexicana de neurociencia':jt OR 'revue internationale de pediatrie':jt OR 'romatizma':jt OR 'sajch south african journal of child health':jt OR 'sleep medicine clinics':jt OR 'south african journal of anaesthesiology and analgesia':jt OR 'southern african journal of anaesthesia and analgesia':jt OR 'transfusion alternatives in transfusion medicine':jt OR 'ultrasound clinics':jt OR 'world heart journal':jt AND [1980-2009]/py

22. ('safety science':jt OR 'salud (i) ciencia':jt OR 'saludarte':jt OR 'salus':jt OR 'sang thrombose vaisseaux':jt OR 'santo tomas journal of medicine':jt OR 'sapporo medical journal':jt OR 'sarcoma':jt OR 'saudi pharmaceutical journal':jt OR 'scandinavian journal of food and nutrition':jt OR 'scandinavian journal of immunology, supplement':jt OR

'scandinavian journal of laboratory animal science':jt OR 'scandinavian journal of work, environment and health, supplement':jt OR 'scandinavian psychoanalytic review':jt OR 'schweizer archiv fur neurologie und psychiatrie':jt OR 'schweizerische zeitschrift fur ganzheitsmedizin':jt OR 'science and sports':jt OR 'scientia pharmaceutica':jt OR 'scientific review of alternative medicine':jt OR 'scripta medica facultatis medicae universitatis brunensis masarykianae':jt OR 'sd revista medica internacional sobre el sindrome de down':jt OR 'seksuologia polska':jt OR 'seleccion':jt OR 'seminarios de la fundacion espanola de reumatologia':jt OR 'seminars in anesthesia, perioperative medicine and pain':jt OR 'seminars in arthroplasty':jt OR 'seminars in breast disease':jt OR 'seminars in cerebrovascular diseases and stroke':jt OR 'seminars in colon and rectal surgery':jt OR 'seminars in hearing':jt OR 'seminars in preventive and alternative medicine':jt OR 'seminars in spine surgery':jt OR 'seminars in surgical oncology':jt OR 'sen':jt OR 'sendrom':jt OR 'sexologies':jt OR 'sexual development':jt OR 'sexuality and disability':jt OR 'sexuologie':jt OR 'signal transduction':jt OR 'simulation in healthcare':jt OR 'sinapse':jt OR 'singapore general hospital proceedings':jt OR 'singapore paediatric journal':jt OR 'skin cancer':jt OR 'skin research':jt OR 'skull base':jt OR 'sleep and biological rhythms':jt OR 'sleep and hypnosis':jt OR 'social cognitive and affective neuroscience':jt OR 'social neuroscience':jt OR 'social work in public health':jt OR 'socijalna psihijatrija':jt OR 'somnologie':jt OR 'south african family practice':jt OR 'south african gastroenterology review':jt OR 'south african journal of clinical nutrition':jt OR 'south african journal of obstetrics and gynaecology':jt OR 'south african journal of psychiatry':jt OR 'southern african journal of critical care':jt OR 'southern african journal of hiv medicine':jt OR 'spectroscopy':jt OR 'stem cell research':jt OR 'stress and health':jt OR 'sucht':jt OR 'suchtmedizin in forschung und praxis':jt OR 'supportive cancer therapy':jt OR 'synlett':jt OR 'synthesis':jt OR 'synthetic communications':jt OR 'taiwan pharmaceutical journal':jt OR 'tanaffos':jt OR 'techniques in foot and ankle surgery':jt OR 'techniques in gastrointestinal endoscopy':jt OR 'techniques in knee surgery':jt AND [1980-2009]/py) OR ('techniques in ophthalmology':jt OR 'techniques in orthopaedics':jt OR 'techniques in regional anesthesia and pain management':jt OR 'technology and disability':jt OR 'tehran university medical journal':jt OR 'teikyo medical journal':jt OR 'terapia psicologica':jt OR 'tetrahedron':jt OR 'tetrahedron asymmetry':jt OR 'tetrahedron letters':jt OR 'thalamus and related systems':jt OR 'therapeutic advances in cardiovascular disease':jt OR 'therapeutic advances in respiratory disease':jt OR 'therapeutic research':jt OR 'therapeutics and clinical risk management':jt OR 'therapy':jt OR 'thermology international':jt OR 'thoracic and cardiovascular surgeon, supplement':jt OR 'thrombosis journal':jt OR 'thyroidology clinical and experimental':jt OR 'tijdschrift voor geneeskunde':jt OR

'tijdschrift voor kindergeneeskunde':jt OR 'tokyo jikeikai medical journal':jt OR 'topics in clinical nutrition':jt OR 'topics in geriatric rehabilitation':jt OR 'topics in spinal cord injury rehabilitation':jt OR 'toxicology international':jt OR 'toxicology mechanisms and methods':jt OR 'toxin reviews':jt OR 'trac - trends in analytical chemistry':jt OR 'trace elements and electrolytes':jt OR 'transfusion medicine and hemotherapy':jt OR 'transfuze a hematologie dnes':jt OR 'translational oncogenomics':jt OR 'transplantation reviews':jt OR 'trasfusione del sangue':jt OR 'trastornos adictivos':jt OR 'trauma':jt OR 'trends in medical research':jt OR 'trends in medicine':jt OR 'trials':jt OR 'tuberculosis and respiratory diseases':jt OR 'tumor':jt OR 'tumor diagnostik und therapie':jt OR 'tumor research':jt OR 'turk geriatri dergisi':jt OR 'turk kardiyoloji dernegi arsivi':jt OR 'turk onkoloji dergisi':jt OR 'turk pediatri arsivi':jt OR 'turk serebrovaskuler hastaliklar dergisi':jt OR 'turkish journal of cancer':jt OR 'turkish journal of hematology':jt OR 'turkish journal of immunology':jt OR 'turkish journal of medical sciences':jt AND [1980-2009]/py) OR ('turkish journal of pharmaceutical sciences':jt OR 'turkiye fiziksel tip ve rehabilitasyon dergisi':jt OR 'turkiye klinikleri journal of medical sciences':jt OR 'tzu chi medical journal':jt OR 'u.s. pharmacist':jt OR 'udar mozgu - problemy interdyscyplinarne':jt OR 'uhod - uluslararasi hematoloji-onkoloji dergisi':jt OR 'ultraschall in der medizin, supplement':jt OR 'ultrasound international':jt OR 'update on cancer therapeutics':jt OR 'urodinamica':jt OR 'vacunas':jt OR 'vascular disease management':jt OR 'vascular disease prevention':jt OR 'vasomed':jt OR 'verdauungskrankheiten':jt OR 'verhaltenstherapie':jt OR 'vigilia sueno':jt OR 'virologica sinica':jt OR 'virologie':jt OR 'visual cognition':jt OR 'visual impairment research':jt OR 'water and environment journal':jt OR 'water science and technology':jt OR 'water, air, and soil pollution':jt OR 'who drug information':jt OR 'wiadomosci psychiatryczne':jt OR 'wideochirurgia i inne techniki maloinwazyjne':jt OR 'wiener klinische wochenschrift, supplement':jt OR 'health':jt OR 'work based learning in primary care':jt OR 'world chinese journal of digestology':jt OR 'world journal of pediatrics':jt OR 'world psychiatry':jt OR 'world wide wounds':jt OR 'wounds uk':jt OR 'wspolczesna onkologia':jt OR 'yakhteh':jt OR 'yeni symposium':jt OR 'yonago acta medica':jt OR 'z zagadnien nauk sadowych':jt OR 'zeitschrift fur allgemeinmedizin':jt OR 'zeitschrift fur epileptologie':jt OR 'zeitschrift fur geburtshilfe und neonatologie, supplement':jt OR 'zeitschrift fur gefassmedizin':jt OR 'zeitschrift fur gerontopsychologie und -psychiatrie':jt OR 'zeitschrift fur orthopadie und unfallchirurgie':jt OR 'zeitschrift fur phytotherapie':jt OR 'zeitschrift fur psychiatrie, psychologie und psychotherapie':jt OR 'zentralsterilisation - central service':jt OR 'allergy, hypersensitivity, asthma':jt OR 'anales de cirugia vascular':jt OR 'anales de patologia vascular':jt OR 'animal technology and welfare':jt OR 'annales de chirurgie

vasculaire':jt OR 'archives of cardiovascular diseases':jt OR 'archives of drug information':jt OR 'archivos de la facultad de medicina de zaragoza':jt OR 'biomedical imaging and intervention journal':jt OR 'blood transfusion':jt OR 'brain stimulation':jt OR 'cbd ubiquitin':jt OR 'child and adolescent psychiatry and mental health':jt OR 'chinese journal of new drugs':jt OR 'clinical and experimental medical letters':jt AND [1980-2009]/py)

23. ('photodiagnosis and photodynamic therapy':jt OR 'physical therapy in sport':jt OR 'physiotherapy':jt OR 'physiotherapy singapore':jt OR 'phytochemistry reviews':jt OR 'phytomedica':jt OR 'phytotherapie':jt OR 'phytotherapie europeenne':jt OR 'piel':jt OR 'pigment cell and melanoma research':jt OR 'plasticidad y restauracion neurologica':jt OR 'plos clinical trials':jt OR 'plos neglected tropical diseases':jt OR 'plos one':jt OR 'polish journal of gynaecological investigations':jt OR 'polish journal of radiology':jt OR 'polski przeglad kardiologiczny':jt OR 'population health metrics':jt OR 'postepy dermatologii i alergologii':jt OR 'postepy mikrobiologii':jt OR 'postepy psychiatrii i neurologii':jt OR 'postepy rehabilitacji':jt OR 'postepy w kardiologii interwencyjnej':jt OR 'postgraduate medicine':jt OR 'powder technology':jt OR 'ppar research':jt OR 'practica pediatrica':jt OR 'practical cardiovascular risk management':jt OR 'practical diabetes international':jt OR 'practical gastroenterology':jt OR 'pravention und rehabilitation':jt OR 'prensa medica argentina':jt OR 'prevention and control':jt OR 'primary care and community psychiatry':jt OR 'primary care companion to the journal of clinical psychiatry':jt OR 'primary psychiatry':jt OR 'problems of infectious and parasitic diseases':jt OR 'proceedings of the controlled release society':jt OR 'progresos en obstetricia y ginecologia':jt OR 'progress in medical research':jt OR 'progress in neurotherapeutics and neuropsychopharmacology':jt OR 'progress in nutrition':jt OR 'progress in palliative care':jt OR 'progress in pediatric cardiology':jt OR 'progressi in reumatologia':jt OR 'proktologia':jt OR 'proteome science':jt OR 'proteomics - clinical applications':jt OR 'przeglad dermatologiczny':jt OR 'przeglad gastroenterologiczny':jt OR 'przeglad menopauzalny':jt OR 'przeglad pediatryczny':jt OR 'psicoterapia cognitiva e comportamentale':jt OR 'psihoterapija':jt OR 'psiquiatria biologica':jt OR 'psiquis':jt OR 'psychiatria i psychologia kliniczna':jt OR 'psychiatric bulletin':jt OR 'psychiatrie':jt OR 'psychiatrische praxis, supplement':jt OR 'psychiatry investigation':jt OR 'psychiatry research - neuroimaging':jt OR 'psychoanalysis and psychotherapy':jt OR 'psychoanalytic psychology':jt OR 'psychoanalytic psychotherapy':jt OR 'psychogeriatria polska':jt OR 'psychogeriatrics':jt OR 'psychoneuro':jt OR 'psycho-oncologie':jt OR 'psychopharmakotherapie':jt OR 'psychoterapia':jt OR 'psychotherapies':jt OR 'psychotherapy':jt OR 'pteridines':jt OR

'public health medicine':jt OR 'publications of takeda research laboratories':jt OR 'puesta al dia en urgencias, emergencias y catastrofes':jt OR 'qatar medical journal':jt OR 'qsar and combinatorial science':jt OR 'quaderni italiani di psichiatria':jt OR 'quality assurance journal':jt OR 'quality in primary care':jt OR 'quimica clinica':jt OR 'radiation physics and chemistry':jt OR 'radiography':jt OR 'radiology and oncology':jt OR 'radiology now':jt OR 'ras - radioloski arhiv srbije':jt OR 'rassegna di patologia':jt OR 'rawal medical journal':jt OR 'reanimation':jt OR 'recent patents on biotechnology':jt OR 'recent patents on dna and gene sequences':jt AND [1980-2009]/py) OR ('recent patents on drug delivery and formulation':jt OR 'recent patents on endocrine, metabolic and immune drug discovery':jt OR 'recent patents on inflammation and allergy drug discovery':jt OR 'rechtsmedizin':jt OR 'records of natural products':jt OR 'rehabilitacion psicosocial':jt OR 'rehabilitation psychology':jt OR 'rentgenologiya i radiologiya':jt OR 'reports of practical oncology and radiotherapy':jt OR 'reproducao e climaterio':jt OR 'reproduction humaine et hormones':jt OR 'reproductive health':jt OR 'reproductive medicine and biology':jt OR 'research and clinical forums':jt OR 'research and practice in alzheimer':jt OR 'research communications in alcohol and substances of abuse':jt OR 'research communications in biochemistry and cell and molecular biology':jt OR 'research communications in biological psychology and psychiatry':jt OR 'research communications in pharmacology and toxicology':jt OR 'research in autism spectrum disorders':jt OR 'research in healthcare financial management':jt OR 'research journal of medical sciences':jt OR 'research journal of pharmacology':jt OR 'resources, conservation and recycling':jt OR 'respiration and circulation':jt OR 'respiratory medicine extra':jt OR 'respiratory medicine':jt OR 'reumatologia clinica':jt OR 'reumatologia clinica suplementos':jt OR 'review of clinical pharmacology and pharmacokinetics, international edition':jt OR 'review of diabetic studies':jt OR 'reviews in analgesia':jt OR 'reviews in clinical and experimental hematology':jt OR 'reviews in clinical gerontology':jt OR 'reviews in contemporary pharmacotherapy':jt OR 'reviews in medical microbiology':jt OR 'reviews on recent clinical trials':jt OR 'revisiones en cancer':jt OR 'revista argentina de dermatologia':jt OR 'revista argentina de endocrinologia y metabolismo':jt OR 'revista brasileira de anestesiologia':jt OR 'revista brasileira de ciencias farmaceuticas/brazilian journal of pharmaceutical sciences':jt OR 'revista brasileira de medicina':jt AND [1980-2009]/py) OR ('revista brasileira de neurologia':jt OR 'revista brasileira de neurologia e psiquiatria':jt OR 'revista brasileira de plantas medicinais':jt OR 'revista brasileira de reumatologia':jt OR 'revista brasileira de toxicologia':jt OR 'revista chilena de neuro-psiquiatria':jt OR 'revista chilena de pediatria':jt OR 'revista de ciencias farmaceuticas basica e aplicada':jt OR 'revista de la sociedad espanola del dolor':jt

OR 'revista de nefrologia, dialisis y trasplante':jt OR 'revista de nutricao':jt OR 'revista de psiquiatria clinica':jt OR 'revista de psiquiatria do rio grande do sul':jt OR 'revista de toxicologia':jt OR 'revista del hospital psiquiatrico de la habana':jt OR 'revista del instituto nacional de enfermedades respiratorias':jt OR 'revista ecuatoriana de neurologia':jt OR 'revista espanola de cardiologia suplementos':jt OR 'revista espanola de enfermedades metabolicas oseas':jt OR 'revista espanola de geriatria y gerontologia':jt OR 'revista espanola de nutricion comunitaria':jt OR 'revista espanola de obesidad':jt OR 'revista espanola de pediatria':jt OR 'revista iberoamericana de fertilidad y reproduccion humana':jt OR 'revista iberoamericana de revisiones en menopausia':jt OR 'revista latinoamericana de hipertension':jt OR 'revista medica de rosario':jt OR 'revista mexicana de anestesiologia':jt OR 'revista mexicana de cardiologia':jt OR 'revista mexicana de ciencias farmaceuticas':jt OR 'revista mexicana de enfermeria cardiologica':jt OR 'revista neurociencias':jt OR 'revista neurologica argentina':jt OR 'revista portuguesa de cardiologia':jt OR 'revista portuguesa de farmacia':jt OR 'revue francaise':jt OR 'rheumatology':jt OR 'ricerca e pratica':jt OR 'ricerca in psicoterapia':jt OR 'rivista di ostetricia e ginecologia':jt OR 'rivista di psichiatria':jt OR 'rivista italiana della medicina di laboratorio':jt OR 'rivista italiana di chirurgia plastica':jt OR 'rivista italiana di medicina':jt OR 'rivista italiana di neurobiologia':jt OR 'rofo fortschritte auf dem gebiet der rontgenstrahlen und der bildgebenden verfahren, supplement':jt OR 'romanian journal of legal medicine':jt OR 'round table series - royal society of medicine':jt OR 's.t.p. pharma pratiques':jt AND [1980-2009]/py)

24. ('krankenhauspharmazie':jt OR 'kumamoto medical journal':jt OR 'kuwait medical journal':jt OR 'laboratoriumsmedizin':jt OR 'laboratory medicine':jt OR 'language and cognitive processes':jt OR 'latin american journal of pharmacy':jt OR 'legal and criminological psychology':jt OR 'letters in drug design and discovery':jt OR 'libri oncologici':jt OR 'lijecnicki vjesnik, supplement':jt OR 'louvain medical':jt OR 'lymphologie in forschung und praxis':jt OR 'malaysian journal of medical sciences':jt OR 'malta medical journal':jt OR 'manufacturing chemist':jt OR 'mapfre medicina':jt OR 'marine drugs':jt OR 'marmara medical journal':jt OR 'matronas profesion':jt OR 'mcgill journal of medicine':jt OR 'measurement in physical education and exercise science':jt OR 'medecine des maladies metaboliques':jt OR 'medecine et chirurgie du pied':jt OR 'medecine et droit':jt OR 'medecine nucleaire':jt OR 'medecine therapeutique':jt OR 'medecine therapeutique - cardio':jt OR 'medical acupuncture':jt OR 'medical crossfire':jt OR 'medical education, supplement':jt OR 'medical forum monthly':jt OR 'medical journal armed forces india':jt OR 'medical journal of minami osaka hospital':jt OR 'medical law international':jt OR 'medical problems of performing artists':jt OR 'medicamundi':jt

OR 'medicina clinica e termale':jt OR 'medicina clinica monografias':jt OR 'medicina cutanea ibero-latino-americana':jt OR 'medicina interna de mexico':jt OR 'medicina paliativa':jt OR 'medicinal chemistry research':jt OR 'medicine today':jt OR 'medicinski glasnik':jt OR 'medico e bambino':jt OR 'medico-legal update':jt OR 'medicus':jt OR 'mediterranean journal of pacing and electrophysiology':jt OR 'medizinische ausbildung':jt OR 'medizinische genetik':jt OR 'medizinische welt':jt OR 'medizintechnik':jt OR 'medycyna paliatywna w praktyce':jt OR 'mental health aspects of developmental disabilities':jt OR 'merec briefing':jt OR 'merec bulletin':jt OR 'merec extra':jt OR 'metabolic syndrome and related disorders':jt OR 'metal-based drugs':jt OR 'methods in cell science':jt OR 'micro and nano letters':jt AND [1980-2009]/py) OR ('microbial cell factories':jt OR 'microbial ecology in health and disease':jt OR 'middle east fertility society journal':jt OR 'middle east journal of emergency medicine':jt OR 'mid-taiwan journal of medicine':jt OR 'mie medical journal':jt OR 'mikologia lekarska':jt OR 'minerva biotecnologica':jt OR 'minerva pneumologica':jt OR 'minerva psichiatrica':jt OR 'minimal invasive chirurgie':jt OR 'modern aspects of immunobiology':jt OR 'molecular oncology':jt OR 'monatsschrift fur kinderheilkunde':jt OR 'mucosal immunology':jt OR 'mutation research - genetic toxicology and environmental mutagenesis':jt OR 'mutation research - reviews in mutation research':jt OR 'nad publication':jt OR 'nadcisnienie tetnicze':jt OR 'nano today':jt OR 'nanobiotechnology':jt OR 'nanoscience':jt OR 'nanotechnology':jt OR 'nanotoxicology':jt OR 'nascer e crescer':jt OR 'natural computing':jt OR 'natural product sciences':jt OR 'nature nanotechnology':jt OR 'ncrp report':jt OR 'ndt plus':jt OR 'nederlands tijdschrift voor anesthesiologie':jt OR 'nederlands tijdschrift voor dermatologie en venereologie':jt OR 'nederlands tijdschrift voor klinische chemie en laboratoriumgeneeskunde':jt OR 'nederlands tijdschrift voor obstetrie en gynaecologie':jt OR 'nervenheilkunde':jt OR 'netherlands heart journal':jt OR 'neural regeneration research':jt OR 'neuroanatomy':jt OR 'neurocomputing':jt OR 'neuroembryology and aging':jt OR 'neuroforum':jt OR 'neurologia croatica':jt OR 'neurologia suplementos':jt OR 'neurologie und rehabilitation':jt OR 'neurology asia':jt OR 'neurology psychiatry and brain research':jt OR 'neurology, neurophysiology and neuroscience':jt OR 'neuromodulation':jt OR 'neuron glia biology':jt OR 'neuro-ophthalmology':jt OR 'neuro-ophthalmology japan':jt OR 'neurophysiologie-labor':jt OR 'neurophysiology':jt OR 'neuropsychiatric disease and treatment':jt OR 'neuropsychiatrie':jt OR 'neuroquantology':jt OR 'neuroradiology journal':jt OR 'neurosciences':jt OR 'neurosurgery quarterly':jt OR 'new genetics and society':jt OR 'new journal of chemistry':jt OR 'new medicine':jt OR 'new zealand journal of medical laboratory science':jt OR 'new zealand public health surveillance report':jt OR 'nieren- und hochdruckkrankheiten':jt OR 'nigerian journal of surgical research':jt OR 'nishinihon

journal of dermatology':jt OR 'nobel medicus':jt OR 'norsk epidemiologi':jt AND [1980-2009]/py) OR ('north and west london journal of general practice':jt OR 'notarzt':jt OR 'nouvelles dermatologiques':jt OR 'nowotwory':jt OR 'nuclear receptor':jt OR 'nutricion clinica y dietetica hospitalaria':jt OR 'nutrition and metabolic disorders in hiv infection':jt OR 'nutrition and metabolism':jt OR 'nutrition clinique et metabolisme':jt OR 'nutrition research':jt OR 'nutrition research reviews':jt OR 'nutritional sciences journal':jt OR 'nutritional therapy and metabolism':jt OR 'obesity research and clinical practice':jt OR 'obstetrics, gynaecology and reproductive medicine':jt OR 'occupational therapy in health care':jt OR 'ochsner journal':jt OR 'oncologia':jt OR 'oncologie':jt OR 'oncology forum':jt OR 'oncology report':jt OR 'onkologe':jt OR 'onkologia polska':jt OR 'operative techniques in general surgery':jt OR 'operative techniques in orthopaedics':jt OR 'operative techniques in otolaryngology - head and neck surgery':jt OR 'operative techniques in sports medicine':jt OR 'operative techniques in thoracic and cardiovascular surgery':jt OR 'oral radiology':jt OR 'oral therapeutics and pharmacology':jt OR 'organogenesis':jt OR 'osteologicky bulletin':jt OR 'osteologie':jt OR 'osteopathische medizin':jt OR 'otjr occupation, participation and health':jt OR 'otorhinolaryngologist':jt OR 'oto-rhino-laryngology tokyo':jt OR 'p and t':jt OR 'padiatrische praxis':jt OR 'paediatria croatica':jt OR 'paediatria croatica, supplement':jt OR 'paediatric and perinatal drug therapy':jt OR 'paediatrics and child health':jt OR 'paediatrics and child health':jt OR 'paediatrics me':jt OR 'pain clinic':jt OR 'pain digest':jt OR 'pakistan journal of medical sciences':jt OR 'pakistan journal of nutrition':jt OR 'pakistan paediatric journal':jt OR 'pan arab journal of neurosurgery':jt OR 'particle and fibre toxicology':jt OR 'particulate science and technology':jt OR 'pat':jt OR 'pathology case reviews':jt OR 'pathophysiology':jt OR 'patologia del aparato locomotor':jt OR 'pediatria catalana':jt OR 'pediatria i medycyna rodzinna':jt OR 'pediatria integral':jt OR 'pediatria polska':jt OR 'pediatria wspolczesna':jt OR 'pediatric asthma, allergy and immunology':jt OR 'pediatric health':jt OR 'pediatrika':jt OR 'perinatology':jt OR 'periodicum biologorum':jt OR 'personalized medicine':jt OR 'perspectives in medicinal chemistry':jt OR 'pharma fokus herz-kreislauf':jt OR 'pharmaca':jt OR 'pharmaceutical biology':jt OR 'pharmaceutical care and research':jt OR 'pharmaceutical care espana':jt OR 'pharmaceutical chemistry journal':jt OR 'pharmaceutical engineering':jt OR 'pharmaceutical journal':jt OR 'pharmaceutical technology':jt OR 'pharmaceutical technology europe':jt OR 'pharmaceuticals':jt OR 'pharmaceutisch weekblad':jt OR 'pharmacia':jt OR 'pharmacien hospitalier':jt OR 'pharmacoeconomics - italian research articles':jt OR 'pharmacoeconomics - spanish research articles':jt OR 'pharmacognosy magazine':jt OR 'pharmacologyonline':jt OR 'pharmacometrics':jt OR 'pharmacy education':jt OR 'pharmacy in practice':jt OR 'pharmacy practice':jt OR 'pharmacy times':jt OR

'pharmakeftiki':jt OR 'pharma-kritik':jt OR 'pharmazeutische industrie':jt OR 'pharmazeutische zeitung':jt OR 'phlebologie':jt OR 'phlebolymphology':jt AND [1980-2009]/py)

25. ('journal of forensic medicine and toxicology':jt OR 'journal of forensic psychiatry and psychology':jt OR 'journal of forensic psychology practice':jt OR 'journal of generic medicines':jt OR 'journal of geriatric cardiology':jt OR 'journal of gynecologic surgery':jt OR 'journal of health science':jt OR 'journal of herbs, spices and medicinal plants':jt OR 'journal of heterocyclic chemistry':jt OR 'journal of histotechnology':jt OR 'journal of hiv/aids prevention in children and youth':jt OR 'journal of hunger and environmental nutrition':jt OR 'journal of immune based therapies and vaccines':jt OR 'journal of immunotoxicology':jt OR 'journal of indian association for child and adolescent mental health':jt OR 'journal of inflammation':jt OR 'journal of intellectual disability research, supplement':jt OR 'journal of internal medicine of india':jt OR 'journal of internal medicine of taiwan':jt OR 'journal of interventional radiology':jt OR 'journal of japanese dental society of anesthesiology':jt OR 'journal of jastro':jt OR 'journal of jilin university medicine edition':jt OR 'journal of korean neurosurgical society':jt OR 'journal of korean society for clinical pharmacology and therapeutics':jt OR 'journal of labelled compounds and radiopharmaceuticals':jt OR 'journal of leukemia and lymphoma':jt OR 'journal of liquid chromatography and related technologies':jt OR 'journal of manual and manipulative therapy':jt OR 'journal of medical and biological engineering':jt OR 'journal of medical biochemistry':jt OR 'journal of medical economics':jt OR 'journal of medical imaging and radiation oncology':jt OR 'journal of medical sciences':jt OR 'journal of medical sciences':jt OR 'journal of medical speech-language pathology':jt OR 'journal of medical teachers federation':jt OR 'journal of medical ultrasonics':jt OR 'journal of medical ultrasound':jt OR 'journal of medicinal plants':jt OR 'journal of medicine':jt OR 'journal of membrane science':jt OR 'journal of men':jt OR 'journal of mental health':jt OR 'journal of mind and behavior':jt OR 'journal of molecular catalysis b: enzymatic':jt OR 'journal of musculoskeletal pain':jt OR 'journal of musculoskeletal research':jt OR 'journal of muslim mental health':jt OR 'journal of nanobiotechnology':jt OR 'journal of nanoparticle research':jt OR 'journal of nara medical association':jt OR 'journal of natural medicines':jt OR 'journal of natural remedies':jt OR 'journal of neonatology':jt AND [1980-2009]/py) OR ('journal of nepal paediatric society':jt OR 'journal of nephrology, urology and transplantation':jt OR 'journal of neurobiology':jt OR 'journal of neurolinguistics':jt OR 'journal of neurological sciences':jt OR 'journal of neurosurgery: pediatrics':jt OR 'journal of neurotherapy':jt OR 'journal of nutrigenetics and nutrigenomics':jt OR 'journal of nutritional

and environmental medicine':jt OR 'journal of oncology practice':jt OR 'journal of organ dysfunction':jt OR 'journal of orthomolecular medicine':jt OR 'journal of orthopaedics and traumatology':jt OR 'journal of outcomes research':jt OR 'journal of pakistan association of dermatologists':jt OR 'journal of pediatric infectious diseases':jt OR 'journal of pediatric neurology':jt OR 'journal of pediatric neurosciences':jt OR 'journal of pediatric urology':jt OR 'journal of pediatrics and neonatology':jt OR 'journal of pelvic medicine and surgery':jt OR 'journal of pharmaceutical finance, economics and policy':jt OR 'journal of pharmaceutical innovation':jt OR 'journal of pharmacology and toxicology':jt OR 'journal of pharmacy of istanbul university':jt OR 'journal of pharmacy practice':jt OR 'journal of pharmacy practice and research':jt OR 'journal of pharmacy technology':jt OR 'journal of phytomedicine and therapeutics':jt OR 'journal of planar chromatography - modern tlc':jt OR 'journal of plastic dermatology':jt OR 'journal of postgraduate medical institute':jt OR 'journal of psychiatry and law':jt OR 'journal of psychopathology and behavioral assessment':jt OR 'journal of psychophysiology':jt OR 'journal of psychotherapy integration':jt OR 'journal of public health':jt OR 'journal of radioanalytical and nuclear chemistry':jt OR 'journal of radiology nursing':jt OR 'journal of radiotherapy in practice':jt OR 'journal of reproductive and infant psychology':jt OR 'journal of research in medical sciences':jt OR 'journal of respiratory diseases':jt OR 'journal of rheumatology and medical rehabilitation':jt OR 'journal of stem cells':jt OR 'journal of substance use':jt OR 'journal of tehran university heart center':jt AND [1980-2009]/py) OR ('journal of the american society of hypertension':jt OR 'journal of the bahrain medical society':jt OR 'journal of the canadian academy of child and adolescent psychiatry':jt OR 'journal of the canadian society of forensic science':jt OR 'journal of the diabetic association of india':jt OR 'journal of the hoffman heart institute of connecticut':jt OR 'journal of the hong kong college of radiologists':jt OR 'journal of the icru':jt OR 'journal of the indian chemical society':jt OR 'journal of the japan epilepsy society':jt OR 'journal of the liaquat university of medical and health sciences':jt OR 'journal of the mechanical behavior of biomedical materials':jt OR 'journal of the osaka city medical center':jt OR 'journal of the royal society for the promotion of health':jt OR 'journal of the royal society of medicine, supplement':jt OR 'journal of the turkish german gynecology association artemis':jt OR 'journal of thermal biology':jt OR 'journal of tokyo medical university':jt OR 'journal of ultrasound':jt OR 'journal of vocational rehabilitation':jt OR 'journal of whiplash and related disorders':jt OR 'jiaotong university (medical sciences)':jt OR 'journal on information technology in healthcare':jt OR 'journal, indian academy of clinical medicine':jt OR 'jpmi - journal of postgraduate medical institute':jt OR

'kardiologia':jt OR 'kardiotechnik':jt OR 'kim - komplementare und integrative medizin, artztezeitschrift fur naturheilverfahren':jt OR 'klinicka biochemie a metabolismus':jt OR 'klinicka imunologia a alergologia':jt OR 'klinicka onkologie':jt OR 'klinik psikofarmakoloji bulteni':jt OR 'klinikarzt':jt OR 'klinische neurophysiologie':jt OR 'koomesh':jt OR 'korean circulation journal':jt OR 'korean journal of cardiovascular diseases':jt OR 'korean journal of medical mycology':jt OR 'korean journal of microbiology and biotechnology':jt OR 'korean journal of pharmacognosy':jt OR 'korean journal of physiology and pharmacology':jt OR 'korean journal of urology':jt OR 'krankenhaushygiene und infektionsverhutung':jt AND [1980-2009]/py)

26. ('international journal of emergency management':jt OR 'international journal of environment and pollution':jt OR 'international journal of essential oil therapeutics':jt OR 'international journal of gerontology':jt OR 'international journal of health promotion and education':jt OR 'international journal of healthcare technology and management':jt OR 'international journal of industrial ergonomics':jt OR 'international journal of intensive care':jt OR 'international journal of leprosy and other mycobacterial diseases':jt OR 'international journal of life cycle assessment':jt OR 'international journal of low radiation':jt OR 'international journal of medical toxicology and legal medicine':jt OR 'international journal of medicinal mushrooms':jt OR 'international journal of medicine':jt OR 'international journal of medicine, biology and the environment':jt OR 'international journal of men':jt OR 'international journal of molecular sciences':jt OR 'international journal of neuroprotection and neuroregeneration':jt OR 'international journal of ophthalmology':jt OR 'international journal of osteopathic medicine':jt OR 'international journal of ozone therapy':jt OR 'international journal of pediatric otorhinolaryngology extra':jt OR 'international journal of peptide research and therapeutics':jt OR 'international journal of pharmaceutical compounding':jt OR 'international journal of pharmacology':jt OR 'international journal of pharmacy practice':jt AND [1980-2009]/py) OR ('international journal of prisoner health':jt OR 'international journal of psychiatry in clinical practice':jt OR 'international journal of respiratory care':jt OR 'international journal of risk and safety in medicine':jt OR 'international journal of sports medicine, supplement':jt OR 'international journal of stroke':jt OR 'international journal of virology':jt OR 'international journal on disability and human development':jt OR 'international medical journal':jt OR 'international pediatrics':jt OR 'international review of allergology and clinical immunology':jt OR 'international seminars in surgical oncology':jt OR 'internet journal of hematology':jt OR 'internista':jt OR 'internistische praxis':jt OR 'interventional neuroradiology':jt OR 'investigacion

cardiovascular':jt OR 'ipcs concise international chemical assessment documents':jt OR 'iranian journal of diabetes and lipid disorders':jt OR 'iranian journal of medical sciences':jt OR 'iranian journal of nuclear medicine':jt OR 'iranian journal of parasitology':jt OR 'iranian journal of pediatrics':jt OR 'iranian journal of pharmaceutical research':jt OR 'iranian journal of pharmacology and therapeutics':jt OR 'iranian journal of radiation research':jt OR 'iranian journal of radiology':jt OR 'iranian red crescent medical journal':jt OR 'irish journal of psychological medicine':jt OR 'isokinetics and exercise science':jt OR 'italian heart journal':jt OR 'italian heart journal supplement':jt OR 'italian heart journal, supplement':jt OR 'italian journal of allergy and clinical immunology':jt OR 'italian journal of gynaecology and obstetrics':jt OR 'italian journal of pediatrics':jt OR 'italian journal of psychiatry and behavioural sciences':jt OR 'italian journal of psychopathology':jt OR 'italian journal of vascular and endovascular surgery':jt OR 'itbm-rbm':jt OR 'itbm-rbm news':jt OR 'jala - journal of the association for laboratory automation':jt OR 'japan medical association journal':jt OR 'japanese journal of chemotherapy':jt OR 'japanese journal of clinical chemistry':jt OR 'japanese journal of clinical pharmacology and therapeutics':jt OR 'japanese journal of clinical radiology':jt OR 'japanese journal of fertility and sterility':jt OR 'japanese journal of interventional cardiology':jt OR 'japanese journal of lung cancer':jt OR 'japanese journal of medical electronics and biological engineering':jt OR 'japanese journal of neurosurgery':jt OR 'japanese pharmacology and therapeutics':jt OR 'java - journal of the association for vascular access':jt OR 'jeur':jt OR 'jinekoloji ve obstetri bulteni':jt OR 'jinekoloji ve obstetrik dergisi':jt OR 'jk practitioner':jt AND [1980-2009]/py) OR ('jk science':jt OR 'jms - journal of medical society':jt OR 'jordan journal of applied sciences - natural sciences':jt OR 'jordan medical journal':jt OR 'jornal brasileiro de psiquiatria':jt OR 'jornal brasileiro de reproducao assistida':jt OR 'jornal vascular brasileiro':jt OR 'journal belge de radiologie':jt OR 'journal de medecine legale droit medical':jt OR 'journal de mycologie medicale':jt OR 'journal de pediatrie et de puericulture':jt OR 'journal de pharmacie clinique':jt OR 'journal de traumatologie du sport':jt OR 'journal for vascular ultrasound':jt OR 'journal fur ernahrungsmedizin':jt OR 'journal fur fertilitat und reproduktion':jt OR 'journal fur gastroenterologische und hepatologische erkrankungen':jt OR 'journal fur hypertonie':jt OR 'journal fur kardiologie':jt OR 'journal fur menopause':jt OR 'journal fur mineralstoffwechsel':jt OR 'journal fur neurologie, neurochirurgie und psychiatrie':jt OR 'journal fur pharmakologie und therapie':jt OR 'journal fur reproduktionsmedizin und endokrinologie':jt OR 'journal international medical sciences academy':jt OR 'journal of aerosol science':jt OR 'journal of aggression, maltreatment and trauma':jt OR 'journal of aging and

pharmacotherapy':jt OR 'journal of anaesthesiology clinical pharmacology':jt OR 'journal of applied biomaterials and biomechanics':jt OR 'journal of applied biomedicine':jt OR 'journal of applied cosmetology':jt OR 'journal of applied gerontology':jt OR 'journal of applied research':jt OR 'journal of atmospheric chemistry':jt OR 'journal of autoimmune diseases':jt OR 'journal of automated methods and management in chemistry':jt OR 'journal of back and musculoskeletal rehabilitation':jt OR 'journal of bacteriology and virology':jt OR 'journal of bioactive and compatible polymers':jt OR 'journal of biolaw and business':jt OR 'journal of biological physics':jt OR 'journal of biological sciences':jt OR 'journal of biomedicine and biotechnology':jt OR 'journal of bodywork and movement therapies':jt OR 'journal of brain science':jt OR 'journal of breath research':jt OR 'journal of bronchology':jt OR 'journal of cancer molecules':jt OR 'journal of cancer research and clinical oncology, supplement':jt OR 'journal of carcinogenesis':jt OR 'journal of cardiothoracic-renal research':jt OR 'journal of cardiovascular computed tomography':jt OR 'journal of cell communication and signaling':jt OR 'journal of chemical technology and biotechnology':jt OR 'journal of child psychotherapy':jt OR 'journal of china pharmaceutical university':jt AND [1980-2009]/py) OR ('journal of chinese medicine':jt OR 'journal of chronic fatigue syndrome':jt OR 'journal of circadian rhythms':jt OR 'journal of clinical and basic cardiology':jt OR 'journal of clinical and diagnostic research':jt OR 'journal of clinical biochemistry and nutrition':jt OR 'journal of clinical dermatology':jt OR 'journal of clinical engineering':jt OR 'journal of clinical ligand assay':jt OR 'journal of clinical lipidology':jt OR 'journal of clinical outcomes management':jt OR 'journal of clinical psychoanalysis':jt OR 'journal of clinical psychology in medical settings':jt OR 'journal of clinical rehabilitative tissue engineering research':jt OR 'journal of clinical research':jt OR 'journal of cognition and development':jt OR 'journal of cognitive psychotherapy':jt OR 'journal of commercial biotechnology':jt OR 'journal of complementary and integrative medicine':jt OR 'journal of contemporary psychotherapy':jt OR 'journal of crohn':jt OR 'colitis supplements':jt OR 'journal of dalian medical university':jt OR 'journal of dermatological science, supplement':jt OR 'journal of diagnostic medical sonography':jt OR 'journal of dietary supplements':jt OR 'journal of drug delivery science and technology':jt OR 'journal of drug evaluation':jt OR 'journal of dual diagnosis':jt OR 'journal of echocardiography':jt OR 'journal of ecophysiology and occupational health':jt OR 'journal of emergency primary health care':jt OR 'journal of emotional abuse':jt OR 'journal of endocrinology, metabolism and diabetes of south africa':jt OR 'journal of epilepsy and clinical neurophysiology':jt OR 'journal of experimental and clinical assisted reproduction':jt OR 'journal of experimental nanoscience':jt OR 'journal of

facial and somato prosthetics':jt OR 'journal of family psychotherapy':jt OR 'journal of food and drug analysis':jt OR 'journal of forensic identification':jt AND [1980-2009]/py)

27. (('bulletin, postgraduate institute of medical education and research, chandigarh':jt OR 'cahiers de nutrition et de dietetique':jt OR 'canadian journal of diabetes':jt OR 'canadian journal of geriatrics':jt OR 'canadian journal of hospital pharmacy':jt OR 'canadian journal of human sexuality':jt OR 'canadian journal of infectious diseases and medical microbiology':jt OR 'canadian journal of respiratory therapy':jt OR 'canadian pharmacists journal':jt OR 'cancer and chemotherapy reviews':jt OR 'cancer cell international':jt OR 'cancer chemotherapy and pharmacology, supplement':jt OR 'cancer forum':jt OR 'cancer informatics':jt OR 'cancer molecular biology':jt OR 'cardiology international':jt OR 'cardiology journal':jt OR 'cardiology review':jt OR 'case reports and clinical practice review':jt OR 'cell and chromosome':jt OR 'cell preservation technology':jt OR 'cell proliferation, supplement':jt OR 'cell stem cell':jt OR 'cells and materials':jt OR 'central european journal of medicine':jt OR 'central nervous system agents in medicinal chemistry':jt OR 'central-european journal of immunology':jt OR 'cephalalgia, supplement':jt OR 'cerebrospinal fluid research':jt OR 'ceska a slovenska gastroenterologie a hepatologie':jt OR 'ceska a slovenska neurologie a neurochirurgie':jt OR 'ceska a slovenska psychiatrie':jt OR 'ceska radiologie':jt OR 'ceska revmatologie':jt OR 'chemometrics and intelligent laboratory systems':jt OR 'chemotherapie journal':jt OR 'chemotherapie journal, supplement':jt OR 'chemtracts':jt OR 'chiba medical journal':jt OR 'child and adolescent mental health':jt OR 'health care':jt OR 'pulmonology':jt OR 'chinese journal of andrology':jt OR 'chinese journal of antibiotics':jt OR 'chinese journal of biologicals':jt OR 'chinese journal of biomedical engineering':jt OR 'chinese journal of cancer biotherapy':jt OR 'chinese journal of cancer prevention and treatment':jt OR 'chinese journal of cancer research':jt OR 'chinese journal of cerebrovascular diseases':jt OR 'chinese journal of clinical nutrition':jt OR 'chinese journal of clinical oncology':jt AND [1980-2009]/py) OR ('chinese journal of contemporary neurology and neurosurgery':jt OR 'chinese journal of endemiology':jt OR 'chinese journal of evidence-based medicine':jt OR 'chinese journal of forensic medicine':jt OR 'chinese journal of gastroenterology':jt OR 'chinese journal of infection and chemotherapy':jt OR 'chinese journal of interventional imaging and therapy':jt OR 'chinese journal of lung cancer':jt OR 'chinese journal of medical imaging technology':jt OR 'chinese journal of microbiology and immunology':jt OR 'chinese journal of natural medicines':jt OR 'chinese journal of neurology':jt OR 'chinese journal of pharmacology and toxicology':jt OR 'chinese journal of radiology':jt OR 'chinese journal of radiology':jt OR 'chinese ophthalmic research':jt OR 'chinese pharmaceutical

journal':jt OR 'chinese pharmacological bulletin':jt OR 'chinese traditional and herbal drugs':jt OR 'chinesische medizin':jt AND [1980-2009]/py) OR ('chiropractic and osteopathy':jt OR 'chirurgia':jt OR 'chirurgia polska':jt OR 'chirurgische gastroenterologie interdisziplinar':jt OR 'chromatographia':jt OR 'ciencia ginecologika':jt OR 'ciencia y tecnologia pharmaceutica':jt OR 'circular farmaceutica':jt OR 'clb chemie in labor und biotechnik':jt OR 'clinica e investigacion en arteriosclerosis':jt OR 'clinica e investigacion en ginecologia y obstetricia':jt OR 'clinical and experimental allergy reviews':jt OR 'clinical and experimental dermatology, supplement':jt OR 'clinical and molecular allergy':jt OR 'clinical and transfusion haematology':jt OR 'clinical application of immunology':jt OR 'clinical case studies':jt OR 'clinical cases in mineral and bone metabolism':jt OR 'clinical dermatology':jt OR 'clinical diabetes':jt OR 'clinical geriatrics':jt OR 'clinical gerontologist':jt OR 'clinical governance':jt OR 'clinical intensive care':jt OR 'clinical kinesiology':jt OR 'clinical microbiology newsletter':jt OR 'clinical mri':jt OR 'clinical neonatology':jt OR 'clinical neuropsychiatry':jt OR 'clinical neuroradiology':jt OR 'clinical nutrition, supplement':jt OR 'clinical pediatric emergency medicine':jt OR 'clinical pediatric endocrinology':jt AND [1980-2009]/py) OR ('clinical practice and epidemiology in mental health':jt OR 'clinical proteomics':jt OR 'clinical psychology and psychotherapy':jt OR 'clinical psychopharmacology and neuroscience':jt OR 'clinical pulmonary medicine':jt OR 'clinical radiology extra':jt OR 'clinical research and regulatory affairs':jt OR 'clinical reviews in bone and mineral metabolism':jt OR 'clinical risk':jt OR 'clinician in management':jt OR 'cme bulletin endocrinology and diabetes':jt OR 'cme cancer medicine':jt OR 'cme journal gastroenterology, hepatology and nutrition':jt OR 'cme journal geriatric medicine':jt OR 'cme journal of gynecologic oncology':jt OR 'cme journal ophthalmology':jt OR 'cme orthopaedics':jt OR 'cocuk cerrahisi dergisi':jt OR 'cocuk sagligi ve hastaliklari dergisi':jt OR 'cognition and emotion':jt OR 'cognitive and behavioral practice':jt OR 'cognitive neurodynamics':jt OR 'cognitive systems research':jt OR 'cognitive therapy and research':jt OR 'colloid and polymer science':jt OR 'colloids and surfaces a':jt OR 'colombia medica':jt OR 'combustion and flame':jt AND [1980-2009]/py) OR ('community eye health journal':jt OR 'community oncology':jt OR 'comparative and functional genomics':jt OR 'comparative biochemistry and physiology':jt OR 'comparative hepatology':jt OR 'computational and mathematical methods in medicine':jt OR 'confinia cephalalgica':jt OR 'confrontations psychiatriques':jt OR 'congenital heart disease':jt OR 'consultant':jt OR 'contemporary hypnosis':jt OR 'continuing medical education - cardiology':jt OR 'continuum lifelong learning in neurology':jt OR 'cor et vasa':jt OR 'core evidence':jt OR 'cosmetic dermatology':jt OR 'cost effectiveness and resource

allocation':jt OR 'cpd anaesthesia':jt OR 'cpd bulletin cellular pathology':jt OR 'cpd bulletin clinical biochemistry':jt OR 'cpd bulletin immunology and allergy':jt OR 'cpd bulletin old age psychiatry':jt OR 'cpd infection':jt OR 'cpd journal radiology update':jt OR 'critical care and shock':jt OR 'critical public health':jt OR 'critical reviews in environmental science and technology':jt OR 'critical reviews in physical and rehabilitation medicine':jt OR 'croatian journal of gastroenterology and hepatology':jt OR 'cuadernos de medicina reproductiva':jt OR 'current anaesthesia and critical care':jt OR 'current bioactive compounds':jt OR 'current bioinformatics':jt AND [1980-2009]/py) OR ('current cancer therapy reviews':jt OR 'current cardiology reviews':jt OR 'current chemical biology':jt OR 'current clinical pharmacology':jt OR 'current computer-aided drug design':jt OR 'current diagnostic pathology':jt OR 'current drug safety':jt OR 'current drug therapy':jt OR 'current enzyme inhibition':jt OR 'current genomics':jt OR 'current hepatitis reports':jt OR 'current hypertension reviews':jt OR 'current immunology reviews':jt OR 'current infectious disease reports':jt OR 'current medical imaging reviews':jt OR 'current medical research and opinion, supplement':jt OR 'current nanoscience':jt OR 'current neuropharmacology':jt OR 'current nutrition and food science':jt OR 'current oncology':jt OR 'current opinion in colloid and interface science':jt OR 'current opinion in hiv and aids':jt OR 'current opinion in organ transplantation':jt OR 'current opinion in orthopaedics':jt OR 'current orthopaedics':jt OR 'current pediatric research':jt OR 'current pediatric reviews':jt OR 'current pharmaceutical analysis':jt OR 'current pharmacogenomics':jt OR 'current prostate reports':jt OR 'current protocols in bioinformatics':jt OR 'current protocols in cytometry':jt OR 'current protocols in human genetics':jt OR 'current protocols in immunology':jt OR 'current protocols in neuroscience':jt OR 'current protocols in nucleic acid chemistry':jt OR 'current protocols in pharmacology':jt OR 'current protocols in protein science':jt OR 'current protocols in stem cell biology':jt OR 'current protocols in toxicology':jt OR 'current psychiatry reviews':jt OR 'current psychosis and therapeutics reports':jt OR 'current respiratory medicine reviews':jt OR 'current rheumatology reviews':jt OR 'current sexual health reports':jt OR 'current signal transduction therapy':jt OR 'current therapeutic research - clinical and experimental':jt OR 'current topics in nutraceutical research':jt OR 'current topics in pharmacology':jt OR 'current treatment options in cardiovascular medicine':jt OR 'current treatment options in gastroenterology':jt OR 'current treatment options in neurology':jt OR 'current trends in immunology':jt OR 'current urology':jt OR 'health reviews':jt OR 'cytojournal':jt OR 'cytopathology, supplement':jt OR 'cytotechnology':jt OR 'daru':jt OR 'debates in neuroscience':jt OR 'dengue bulletin':jt OR 'deri hastaliklari ve frengi arsivi':jt OR 'dermatologia clinica':jt OR 'dermatologia kliniczna':jt OR

'dermatologia revista mexicana':jt OR 'dermatologica sinica':jt OR 'dermatologie in beruf und umwelt':jt OR 'deutsche apotheker zeitung':jt OR 'deutsche zeitschrift fur akupunktur':jt OR 'deutsche zeitschrift fur onkologie':jt OR 'deutsches arzteblatt':jt OR 'dhaka university journal of pharmaceutical sciences':jt OR 'diabetes and metabolic syndrome':jt OR 'diabetes spectrum':jt OR 'diabetes, obesity and metabolism, supplement':jt OR 'diabetes, stoffwechsel und herz':jt OR 'diabetologia croatica':jt OR 'diabetologia doswiadczalna i kliniczna':jt OR 'diabetologia polska':jt OR 'diabetologie metabolismus endokrinologie vyziva':jt OR 'dialisis y trasplante':jt OR 'dialogues in cardiovascular medicine':jt OR 'dialysis and transplantation':jt OR 'digestive endoscopy':jt OR 'disease management and health outcomes':jt OR 'diving and hyperbaric medicine':jt OR 'dokkyo journal of medical sciences':jt AND [1980-2009]/py)) OR (('european journal of endocrinology, supplement':jt OR 'european journal of general medicine':jt OR 'european journal of heart failure, supplement':jt OR 'european journal of herbal medicine':jt OR 'european journal of inflammation':jt OR 'european journal of internal medicine':jt OR 'european journal of lymphology and related problems':jt OR 'european journal of oncology':jt OR 'european journal of oriental medicine':jt OR 'european journal of orthopaedic surgery and traumatology':jt OR 'european journal of pediatric dermatology':jt OR 'european journal of pediatric surgery, supplement':jt OR 'european journal of pediatrics, supplement':jt OR 'european journal of plastic surgery':jt OR 'european journal of psychiatry':jt OR 'european journal of special needs education':jt OR 'european pharmaceutical contractor':jt OR 'european radiology, supplement':jt OR 'european respiratory review':jt OR 'european surgery - acta chirurgica austriaca':jt OR 'european urology, supplements':jt OR 'evidence-based communication assessment and intervention':jt OR 'evidence-based complementary and alternative medicine':jt OR 'evidence-based gastroenterology':jt OR 'evidence-based ophthalmology':jt OR 'evolution psychiatrique':jt OR 'evolutionary bioinformatics':jt OR 'evrs educational electronic journal':jt OR 'experimental and clinical cardiology':jt OR 'experimental and clinical hepatology':jt OR 'expert opinion on drug discovery':jt OR 'expert opinion on medical diagnostics':jt OR 'expert opinion on therapeutic patents':jt OR 'expert review of clinical immunology':jt OR 'expert review of clinical pharmacology':jt OR 'expert review of dermatology':jt OR 'expert review of endocrinology and metabolism':jt OR 'expert review of gastroenterology and hepatology':jt OR 'expert review of obstetrics and gynecology':jt OR 'expert review of ophthalmology':jt OR 'expert review of pharmacoeconomics and outcomes research':jt OR 'expert review of respiratory medicine':jt OR 'fabad journal of pharmaceutical sciences':jt OR 'facts, news and views':jt OR 'families, systems and health':jt OR 'family medicine and primary care review':jt OR 'farmaceuticky obzor':jt OR 'farmaceutico hospitales':jt OR

'farmaceutisch tijdschrift voor belgie':jt AND [1980-2009]/py) OR ('farmaceutski glasnik':jt OR 'farmacevtski vestnik':jt OR 'farmacia':jt OR 'fetal and maternal medicine review':jt OR 'feuillets de radiologie':jt OR 'fizjoterapia':jt OR 'fizjoterapia polska':jt OR 'fizyoterapi rehabilitasyon':jt OR 'flavour and fragrance journal':jt OR 'fluoride':jt OR 'fmc formacion medica continuada en atencion primaria':jt OR 'focus on alternative and complementary therapies':jt OR 'focus on parkinson':jt OR 'food chemistry':jt OR 'foot':jt OR 'foot and ankle surgery':jt OR 'forensic science international: genetics':jt OR 'forensic toxicology':jt OR 'formulary':jt OR 'foundation years':jt OR 'frontiers in drug design and discovery':jt OR 'frontiers in medicinal chemistry':jt OR 'future cardiology':jt OR 'future hiv therapy':jt OR 'future lipidology':jt OR 'future neurology':jt OR 'future rheumatology':jt OR 'future virology':jt OR 'gastric and breast cancer':jt OR 'gastroenterologia polska':jt OR 'gastroenterological endoscopy':jt OR 'gastroenterology and hepatology':jt OR 'gastroenterology international':jt OR 'gazi medical journal':jt OR 'gazi universitesi eczacilik fakultesi dergisi':jt OR 'gazzetta medica italiana archivio per le scienze mediche':jt AND [1980-2009]/py) OR ('geburtshilfe und frauenheilkunde':jt OR 'ged - gastrenterologia endoscopia digestiva':jt OR 'gefahrstoffe reinhaltung der luft':jt OR 'gefasschirurgie':jt OR 'gender and psychoanalysis':jt OR 'gene regulation and systems biology':jt OR 'gene therapy and regulation':jt OR 'geneesmiddelenbulletin':jt OR 'general medicine':jt OR 'genes and nutrition':jt OR 'genetic vaccines and therapy':jt OR 'genetics and molecular biology':jt OR 'genome dynamics':jt OR 'geriatric and medical intelligence':jt OR 'geriatrika':jt OR 'german journal of psychiatry':jt OR 'gerokomos':jt OR 'gesundheitsokonomie und qualitatsmanagement':jt OR 'gesundheitswesen, supplement':jt OR 'gimt - giornale italiano delle malattie del torace':jt OR 'ginecologia y obstetricia clinica':jt OR 'ginekologia onkologiczna':jt OR 'ginekologia praktyczna':jt OR 'giornale di gerontologia':jt OR 'giornale italiano di dermatologia e venereologia':jt OR 'giornale italiano di diabetologia e metabolismo':jt OR 'giornale italiano di endoscopia digestiva':jt OR 'giornale italiano di farmacia clinica':jt OR 'giornale italiano di medicina tropicale':jt OR 'giornale italiano di ostetricia e ginecologia':jt OR 'global public health':jt OR 'globalization and health':jt OR 'gogus-kalp-damar anestezi ve yogun bakim dernegi dergisi':jt OR 'good clinical practice journal':jt OR 'gynaecologia et perinatologia':jt OR 'gynaecologia et perinatologia, supplement':jt OR 'gynakologe':jt OR 'gynakologie':jt OR 'gynakologische endokrinologie':jt AND [1980-2009]/py) OR ('gynakologische praxis':jt OR 'gynecological surgery':jt OR 'haema':jt OR 'haematologica reports':jt OR 'hallym international journal of aging':jt OR 'harm reduction journal':jt OR 'haut':jt OR 'headache and pain':jt OR 'health economics, policy and law':jt

OR 'health education journal':jt OR 'health research policy and systems':jt OR 'health services and outcomes research methodology':jt OR 'health, risk and society':jt OR 'heart and metabolism':jt OR 'helicobacter, supplement':jt OR 'helvetica chimica acta':jt OR 'hematologie':jt OR 'hepatitis monthly':jt OR 'hepato-gastro':jt OR 'hepatologia polska':jt OR 'hepatology international':jt OR 'hepatology research':jt OR 'hereditary cancer in clinical practice':jt OR 'heroin addiction and related clinical problems':jt OR 'herz, supplement':jt OR 'high blood pressure and cardiovascular prevention':jt OR 'hip international':jt OR 'hipertension':jt OR 'hippokratia':jt OR 'hispanic health care international':jt OR 'hiv and aids review':jt AND [1980-2009]/py) OR ('homeostasis in health and disease':jt OR 'hong kong journal of dermatology and venereology':jt OR 'hong kong journal of emergency medicine':jt OR 'hong kong journal of nephrology':jt OR 'hong kong journal of occupational therapy':jt OR 'hong kong journal of paediatrics':jt OR 'hong kong journal of psychiatry':jt OR 'hong kong physiotherapy journal':jt OR 'hong kong practitioner':jt OR 'hormone and metabolic research, supplement':jt OR 'hospital pharmacist':jt OR 'hospital pharmacy':jt OR 'hpb':jt OR 'huisarts en wetenschap':jt OR 'human development':jt OR 'human movement':jt OR 'iatreia':jt OR 'ijci - international journal of clinical investigation':jt OR 'imaging':jt OR 'imaging decisions mri':jt OR 'imago':jt OR 'immunity and ageing':jt OR 'immuno-analyse et biologie specialisee':jt OR 'immunologiya':jt OR 'immunology, endocrine and metabolic agents in medicinal chemistry':jt OR 'impegno ospedaliero, sezione scientifica':jt OR 'indian drugs':jt OR 'indian journal of biotechnology':jt OR 'indian journal of clinical biochemistry':jt OR 'indian journal of critical care medicine':jt OR 'indian journal of dermatology':jt OR 'indian journal of hematology and blood transfusion':jt OR 'indian journal of human genetics':jt OR 'indian journal of medical research, supplement':jt OR 'indian journal of occupational and environmental medicine':jt OR 'indian journal of pharmaceutical sciences':jt AND [1980-2009]/py) OR ('indian journal of pharmacology':jt OR 'indian journal of practical pediatrics':jt OR 'indian journal of radiology and imaging':jt OR 'indian journal of rheumatology':jt OR 'indian pacing and electrophysiology journal':jt OR 'indoor and built environment':jt OR 'infancy':jt OR 'infant mental health journal':jt AND [1980-2009]/py) OR ('infanto':jt OR 'infection, supplement':jt OR 'infections in medicine':jt OR 'infectious agents and cancer':jt OR 'infectious diseases in clinical practice':jt OR 'infektoloski glasnik':jt OR 'inmunologia':jt OR 'innovations in pharmaceutical technology':jt OR 'technology and techniques in cardiothoracic and vascular surgery':jt OR 'insulin':jt OR 'integrative medicine insights':jt AND [1980-2009]/py) OR ('notfallbehandlung':jt OR 'internal medicine clinical and laboratory':jt OR 'international forum of psychoanalysis':jt OR 'international journal for equity in health':jt OR

'international journal of andrology, supplement':jt OR 'international journal of angiology':jt OR 'international journal of atherosclerosis':jt OR 'international journal of behavioral development':jt OR 'international journal of behavioral nutrition and physical activity':jt OR 'international journal of biomedical science':jt OR 'international journal of biostatistics':jt OR 'international journal of biotechnology':jt OR 'international journal of cancer prevention':jt OR 'international journal of cancer research':jt OR 'international journal of computer assisted radiology and surgery':jt OR 'international journal of cosmetic science':jt OR 'international journal of diabetes and metabolism':jt OR 'international journal of disaster medicine':jt AND [1980-2009]/py))

28. ('academic journal of second military medical university':jt OR 'jiaotong university':jt OR 'acc cardiosource review journal':jt OR 'acta anaesthesiologica italica / anaesthesia and intensive care in italy':jt OR 'acta anatomica sinica':jt OR 'acta angiologica':jt OR 'acta biologica szegediensis':jt OR 'acta cardiologica sinica':jt OR 'acta clinica croatica':jt OR 'acta clinica croatica, supplement':jt OR 'acta criminologiae et medicinae legalis japonica':jt OR 'acta endoscopica':jt OR 'acta facultatis medicae fluminensis':jt OR 'acta ginecologica':jt OR 'acta haematologica polonica':jt OR 'acta hepatologica japonica':jt OR 'acta histochemica et cytochemica':jt OR 'acta hospitalia':jt OR 'acta medica austriaca':jt OR 'acta medica auxologica':jt OR 'acta medica bulgarica':jt OR 'acta medica et biologica':jt OR 'acta medica iranica':jt OR 'acta medica mediterranea':jt OR 'acta medica nagasakiensia':jt OR 'acta medica romana':jt OR 'acta microbiologica hellenica':jt OR 'acta neuropsychiatrica':jt OR 'acta neuropsychologica':jt OR 'acta pediatrica espanola':jt OR 'acta pharmaceutica sciencia':jt OR 'acta scientiarum - biological sciences':jt OR 'acta scientiarum - health sciences':jt OR 'acta toxicologica':jt OR 'actualites pharmaceutiques':jt OR 'actualites pharmaceutiques hospitalieres':jt OR 'actualizaciones en anestesiologia y reanimacion':jt OR 'acute medicine':jt OR 'acute pain':jt OR 'addictive disorders and their treatment':jt OR 'adipocytes':jt OR 'advances in breast cancer':jt OR 'advances in clinical and experimental medicine':jt OR 'advances in gastrointestinal cancers':jt OR 'advances in mind-body medicine':jt OR 'advances in pharmacological sciences':jt OR 'advances in pharmacy':jt OR 'advances in physiotherapy':jt OR 'advances in psychiatric treatment':jt OR 'adverse drug reaction bulletin':jt OR 'aerobiologia':jt OR 'aerosol science and technology':jt OR 'aesthetic surgery journal':jt OR 'african journal biomedical research':jt OR 'african journal of aids research':jt OR 'african journal of biotechnology':jt OR 'african journal of neurological sciences':jt OR 'african journal of psychiatry':jt OR 'african journal of traditional, complementary and alternative medicines':jt OR 'age':jt OR 'aggression and violent behavior':jt OR 'aging health':jt OR 'aids research and

therapy':jt OR 'aktuality v nefrologii':jt OR 'aktualnosci neurologiczne':jt OR 'aktuelle dermatologie':jt OR 'aktuelle ernahrungsmedizin':jt OR 'aktuelle neurologie':jt OR 'aktuelle neurologie, supplement':jt OR 'aktuelle rheumatologie':jt OR 'aktuelle rheumatologie, supplement':jt OR 'aktuelle traumatologie, supplement':jt OR 'akupunktur und traditionelle chinesische medizin':jt OR 'alcoholism':jt OR 'alcoholism treatment quarterly':jt OR 'alergia astma immunologia':jt OR 'alergie':jt OR 'alergologia e inmunologia clinica':jt OR 'alimentary pharmacology and therapeutics symposium series':jt OR 'allergo journal':jt OR 'allergologie':jt OR 'allergy and clinical immunology international':jt OR 'allergy, asthma and clinical immunology':jt OR 'alter':jt OR 'dementia':jt OR 'ambulatory surgery':jt OR 'american journal of biochemistry and biotechnology':jt OR 'american journal of forensic psychiatry':jt OR 'american journal of forensic psychology':jt OR 'american journal of hematology/ oncology':jt OR 'american journal of immunology':jt OR 'american journal of infectious diseases':jt OR 'american journal of pharmacology and toxicology':jt OR 'american journal of psychiatric rehabilitation':jt OR 'american pharmaceutical outsourcing':jt OR 'american pharmaceutical review':jt OR 'anadolu psikiyatri dergisi':jt OR 'anae - approche neuropsychologique des apprentissages':jt OR 'anaesthesia and intensive care medicine':jt OR 'anaesthesiology and intensive care':jt OR 'anais brasileiros de dermatologia':jt OR 'anales de pediatria monografias':jt AND [1980-2009]/py) OR ('anales de psiquiatria':jt OR 'anasthesiologie intensivmedizin notfallmedizin schmerztherapie, supplement':jt OR 'anasthesiologie und intensivmedizin':jt OR 'anatolian journal of clinical investigation':jt OR 'anatomical sciences education':jt OR 'anestesia en mexico':jt OR 'anestezi dergisi':jt OR 'anesteziologie a intenzivni medicina':jt OR 'anestezjologia intensywna terapia':jt OR 'anesthesia and resuscitation':jt OR 'anesthesiology research and practice':jt OR 'angeiologie':jt OR 'angiologia':jt OR 'internet journal of forensic medicine and toxicology':jt OR 'animal':jt OR 'animal welfare':jt OR 'ankara universitesi eczacilik fakultesi dergisi':jt OR 'annales medico-psychologiques':jt OR 'annales nestle':jt OR 'annali italiani di dermatologia allergologica clinica e sperimentale':jt OR 'annali italiani di medicina interna':jt OR 'annals of cancer research and therapy':jt OR 'annals of gastroenterology':jt OR 'annals of general psychiatry':jt OR 'annals of hematology, supplement':jt OR 'annals of indian academy of neurology':jt OR 'annals of long-term care':jt OR 'annals of medical sciences':jt OR 'annals of thoracic medicine':jt OR 'annexins':jt OR 'annual report of shionogi research laboratory':jt OR 'annual review of biomedical sciences':jt OR 'anthropology and medicine':jt OR 'antibiotiques':jt OR 'anti-infective agents in medicinal chemistry':jt OR 'anti-inflammatory and anti-allergy agents in medicinal chemistry':jt OR 'anuario de psicologia':jt OR 'anz

nuclear medicine':jt OR 'aphasiology':jt OR 'aplar journal of rheumatology':jt OR 'applied cardiopulmonary pathophysiology':jt OR 'applied cognitive psychology':jt OR 'applied radiology':jt OR 'apunts medicine':jt OR 'archive of oncology':jt OR 'archives of dermatological research, supplement':jt OR 'archives of gastroenterohepatology':jt OR 'archives of hellenic medicine':jt OR 'archives of medical science':jt OR 'archives of osteoporosis':jt OR 'archives of psychiatry and psychotherapy':jt OR 'archives of the balkan medical union':jt OR 'archivio di ostetricia e ginecologia':jt OR 'archivos de medicina':jt OR 'archivos de neurociencias':jt OR 'archivos de psiquiatria':jt OR 'archivos venezolanos de farmacologia y terapeutica':jt OR 'argomenti di gastroenterologia clinica':jt OR 'arquivos brasileiros de medicina':jt OR 'arquivos de geriatria e gerontologia':jt OR 'arquivos de medicina':jt OR 'ars pharmaceutica':jt OR 'artery research':jt OR 'arts in psychotherapy':jt OR 'arzneimitteltherapie':jt OR 'asge clinical update':jt OR 'asia pacific journal of environmental law':jt OR 'asian journal of microbiology, biotechnology and environmental sciences':jt OR 'asian journal of oral and maxillofacial surgery':jt OR 'asian oceanian journal of radiology':jt AND [1980-2009]/py) OR ('asia-pacific journal of clinical oncology':jt OR 'asthma journal':jt OR 'atemwegs- und lungenkrankheiten':jt OR 'atencion farmaceutica':jt OR 'athletic therapy today':jt OR 'atmospheric environment':jt OR 'audiological medicine':jt OR 'australasian biotechnology':jt OR 'australia and new zealand health policy':jt OR 'australian journal of forensic sciences':jt OR 'australian journal of medical science':jt OR 'australian journal of pharmacy':jt OR 'australian journal of primary health':jt OR 'australian prescriber':jt OR 'awic bulletin':jt OR 'azerbaijan medical journal':jt OR 'bag - journal of basic and applied genetics':jt OR 'bahrain medical bulletin':jt OR 'balkan journal of medical genetics':jt OR 'bangladesh journal of dermatology, venereology and leprology':jt OR 'bangladesh journal of obstetrics and gynecology':jt OR 'bangladesh renal journal':jt OR 'basic and clinical pharmacology and toxicology, supplement':jt OR 'behavioral and brain functions':jt OR 'behaviour change':jt OR 'behavioural and cognitive psychotherapy':jt OR 'best practice and research in clinical anaesthesiology':jt OR 'biocatalysis and biotransformation':jt OR 'biochemia medica':jt OR 'biochemical engineering journal':jt OR 'biochemistry, supplemental series a':jt OR 'biochemistry, supplemental series b':jt OR 'biofilms':jt OR 'biogenic amines':jt OR 'biological procedures online':jt OR 'biological rhythm research':jt OR 'biologische medizin':jt OR 'biomarker insights':jt OR 'biomarkers in medicine':jt OR 'biomedical engineering':jt OR 'biomedical engineering - applications, basis and communications':jt OR 'biomedical materials':jt OR 'biomedical research':jt OR 'biomedical reviews':jt OR 'biomedical signal processing and control':jt OR 'biomedicine':jt OR 'biomolecular engineering':jt OR 'biopharm international':jt OR 'biophysical

reviews and letters':jt OR 'biosciences biotechnology research asia':jt OR 'biotechnology and bioprocess engineering':jt OR 'biotecnologia aplicada':jt OR 'biotherapy':jt OR 'bipolar disorders, supplement':jt OR 'bju international, supplement':jt OR 'blutalkohol':jt OR 'bmc anesthesiology':jt OR 'bmc blood disorders':jt OR 'bmc chemical biology':jt OR 'bmc clinical pathology':jt OR 'bmc ear, nose and throat disorders':jt OR 'bmc emergency medicine':jt OR 'bmc endocrine disorders':jt OR 'bmc international health and human rights':jt OR 'bmc news and views':jt OR 'bmc nuclear medicine':jt OR 'bmc palliative care':jt OR 'body, movement and dance in psychotherapy':jt OR 'bolest':jt OR 'bollettino - lega italiana contro':jt OR 'breast cancer online':jt OR 'breast care':jt OR 'bridging eastern and western psychiatry':jt OR 'british journal of cardiology':jt OR 'british journal of clinical pharmacology, supplement':jt OR 'british journal of dermatology, supplement':jt OR 'british journal of developmental disabilities':jt OR 'british journal of diabetes and vascular disease':jt OR 'british journal of intensive care':jt OR 'british journal of psychotherapy':jt OR 'bulgarian medicine':jt OR 'bull rgkmc':jt OR 'bulletin of the chemical society of japan':jt AND [1980-2009]/py) OR ('bulletin, postgraduate institute of medical education and research, chandigarh':jt OR 'cahiers de nutrition et de dietetique':jt OR 'canadian journal of diabetes':jt OR 'canadian journal of geriatrics':jt OR 'canadian journal of hospital pharmacy':jt OR 'canadian journal of human sexuality':jt OR 'canadian journal of infectious diseases and medical microbiology':jt OR 'canadian journal of respiratory therapy':jt OR 'canadian pharmacists journal':jt OR 'cancer and chemotherapy reviews':jt OR 'cancer cell international':jt OR 'cancer chemotherapy and pharmacology, supplement':jt OR 'cancer forum':jt OR 'cancer informatics':jt OR 'cancer molecular biology':jt OR 'cardiology international':jt OR 'cardiology journal':jt OR 'cardiology review':jt OR 'case reports and clinical practice review':jt OR 'cell and chromosome':jt OR 'cell preservation technology':jt OR 'cell proliferation, supplement':jt OR 'cell stem cell':jt OR 'cells and materials':jt OR 'central european journal of medicine':jt OR 'central nervous system agents in medicinal chemistry':jt OR 'central-european journal of immunology':jt OR 'cephalalgia, supplement':jt OR 'cerebrospinal fluid research':jt OR 'ceska a slovenska gastroenterologie a hepatologie':jt OR 'ceska a slovenska neurologie a neurochirurgie':jt OR 'ceska a slovenska psychiatrie':jt OR 'ceska radiologie':jt OR 'ceska revmatologie':jt OR 'chemometrics and intelligent laboratory systems':jt OR 'chemotherapie journal':jt OR 'chemotherapie journal, supplement':jt OR 'chemtracts':jt OR 'chiba medical journal':jt OR 'child and adolescent mental health':jt OR 'health care':jt OR 'pulmonology':jt OR 'chinese journal of andrology':jt OR 'chinese journal of antibiotics':jt OR 'chinese journal of biologicals':jt OR 'chinese journal of biomedical engineering':jt OR 'chinese journal of cancer biotherapy':jt OR

'chinese journal of cancer prevention and treatment':jt OR 'chinese journal of cancer research':jt OR 'chinese journal of cerebrovascular diseases':jt OR 'chinese journal of clinical nutrition':jt OR 'chinese journal of clinical oncology':jt AND [1980-2009]/py)

29. 'academic journal of second military medical university':jt OR 'jiaotong university':jt OR 'acc cardiosource review journal':jt OR 'acta anaesthesiologica italica / anaesthesia and intensive care in italy':jt OR 'acta anatomica sinica':jt OR 'acta angiologica':jt OR 'acta biologica szegediensis':jt OR 'acta cardiologica sinica':jt OR 'acta clinica croatica':jt OR 'acta clinica croatica, supplement':jt OR 'acta criminologiae et medicinae legalis japonica':jt OR 'acta endoscopica':jt OR 'acta facultatis medicae fluminensis':jt OR 'acta ginecologica':jt OR 'acta haematologica polonica':jt OR 'acta hepatologica japonica':jt OR 'acta histochemica et cytochemica':jt OR 'acta hospitalia':jt OR 'acta medica austriaca':jt OR 'acta medica auxologica':jt OR 'acta medica bulgarica':jt OR 'acta medica et biologica':jt OR 'acta medica iranica':jt OR 'acta medica mediterranea':jt OR 'acta medica nagasakiensia':jt OR 'acta medica romana':jt OR 'acta microbiologica hellenica':jt OR 'acta neuropsychiatrica':jt OR 'acta neuropsychologica':jt OR 'acta pediatrica espanola':jt OR 'acta pharmaceutica sciencia':jt OR 'acta scientiarum - biological sciences':jt OR 'acta scientiarum - health sciences':jt OR 'acta toxicologica':jt OR 'actualites pharmaceutiques':jt OR 'actualites pharmaceutiques hospitalieres':jt OR 'actualizaciones en anestesiologia y reanimacion':jt OR 'acute medicine':jt OR 'acute pain':jt OR 'addictive disorders and their treatment':jt OR 'adipocytes':jt OR 'advances in breast cancer':jt OR 'advances in clinical and experimental medicine':jt OR 'advances in gastrointestinal cancers':jt OR 'advances in mind-body medicine':jt OR 'advances in pharmacological sciences':jt OR 'advances in pharmacy':jt OR 'advances in physiotherapy':jt OR 'advances in psychiatric treatment':jt OR 'adverse drug reaction bulletin':jt OR 'aerobiologia':jt OR 'aerosol science and technology':jt OR 'aesthetic surgery journal':jt OR 'african journal biomedical research':jt OR 'african journal of aids research':jt OR 'african journal of biotechnology':jt OR 'african journal of neurological sciences':jt OR 'african journal of psychiatry':jt OR 'african journal of traditional, complementary and alternative medicines':jt OR 'age':jt OR 'aggression and violent behavior':jt OR 'aging health':jt OR 'aids research and therapy':jt OR 'aktuality v nefrologii':jt OR 'aktualnosci neurologiczne':jt OR 'aktuelle dermatologie':jt OR 'aktuelle ernahrungsmedizin':jt OR 'aktuelle neurologie':jt OR 'aktuelle neurologie, supplement':jt OR 'aktuelle rheumatologie':jt OR 'aktuelle rheumatologie, supplement':jt OR 'aktuelle traumatologie, supplement':jt OR 'akupunktur und traditionelle chinesische medizin':jt OR 'alcoholism':jt OR 'alcoholism treatment quarterly':jt OR 'alergia astma immunologia':jt OR

'alergie':jt OR 'alergologia e inmunologia clinica':jt OR 'alimentary pharmacology and therapeutics symposium series':jt OR 'allergo journal':jt OR 'allergologie':jt OR 'allergy and clinical immunology international':jt OR 'allergy, asthma and clinical immunology':jt OR 'alter':jt OR 'dementia':jt OR 'ambulatory surgery':jt OR 'american journal of biochemistry and biotechnology':jt OR 'american journal of forensic psychiatry':jt OR 'american journal of forensic psychology':jt OR 'american journal of hematology/ oncology':jt OR 'american journal of immunology':jt OR 'american journal of infectious diseases':jt OR 'american journal of pharmacology and toxicology':jt OR 'american journal of psychiatric rehabilitation':jt OR 'american pharmaceutical outsourcing':jt OR 'american pharmaceutical review':jt OR 'anadolu psikiyatri dergisi':jt OR 'anae - approche neuropsychologique des apprentissages chez':jt OR 'anaesthesia and intensive care medicine':jt OR 'anaesthesiology and intensive care':jt OR 'anais brasileiros de dermatologia':jt OR 'anales de pediatria monografias':jt OR 'anales de psiquiatria':jt OR 'anasthesiologie intensivmedizin notfallmedizin schmerztherapie, supplement':jt OR 'anasthesiologie und intensivmedizin':jt OR 'anatolian journal of clinical investigation':jt OR 'anatomical sciences education':jt OR 'anestesia en mexico':jt OR 'anestezi dergisi':jt OR 'anesteziologie a intenzivni medicina':jt OR 'anestezjologia intensywna terapia':jt OR 'anesthesia and resuscitation':jt OR 'anesthesiology research and practice':jt OR 'angeiologie':jt OR 'angiologia':jt OR 'internet journal of forensic medicine and toxicology':jt OR 'animal':jt OR 'animal welfare':jt OR 'ankara universitesi eczacilik fakultesi dergisi':jt OR 'annales medico-psychologiques':jt OR 'annales nestle':jt OR 'annali italiani di dermatologia allergologica clinica e sperimentale':jt OR 'annali italiani di medicina interna':jt OR 'annals of cancer research and therapy':jt OR 'annals of gastroenterology':jt OR 'annals of general psychiatry':jt OR 'annals of hematology, supplement':jt OR 'annals of indian academy of neurology':jt OR 'annals of long-term care':jt OR 'annals of medical sciences':jt OR 'annals of thoracic medicine':jt OR 'annexins':jt OR 'annual report of shionogi research laboratory':jt OR 'annual review of biomedical sciences':jt OR 'anthropology and medicine':jt OR 'antibiotiques':jt OR 'anti-infective agents in medicinal chemistry':jt OR 'anti-inflammatory and anti-allergy agents in medicinal chemistry':jt OR 'anuario de psicologia':jt OR 'anz nuclear medicine':jt OR 'aphasiology':jt OR 'aplar journal of rheumatology':jt OR 'applied cardiopulmonary pathophysiology':jt OR 'applied cognitive psychology':jt OR 'applied radiology':jt OR 'apunts medicine':jt OR 'archive of oncology':jt OR 'archives of dermatological research, supplement':jt OR 'archives of gastroenterohepatology':jt OR 'archives of hellenic medicine':jt OR 'archives of medical science':jt OR 'archives of osteoporosis':jt OR 'archives of psychiatry and psychotherapy':jt OR 'archives of the balkan medical union':jt

```
OR 'archivio di ostetricia e ginecologia':jt OR 'archivos de
medicina':jt OR 'archivos de neurociencias':jt OR 'archivos de
psiquiatria':jt OR 'archivos venezolanos de farmacologia y
terapeutica':jt OR 'argomenti di gastroenterologia clinica':jt
OR 'arquivos brasileiros de medicina':jt OR 'arquivos de
geriatria e gerontologia':jt OR 'arquivos de medicina':jt OR
'ars pharmaceutica':jt OR 'artery research':jt OR 'arts in
psychotherapy':jt OR 'arzneimitteltherapie':jt OR 'asge clinical
update':jt OR 'asia pacific journal of environmental law':jt OR
'asian journal of microbiology, biotechnology and environmental
sciences':jt OR 'asian journal of oral and maxillofacial
surgery':jt OR 'asian oceanian journal of radiology':jt OR
'asia-pacific journal of clinical oncology':jt OR 'asthma
journal':jt OR 'atemwegs- und lungenkrankheiten':jt OR 'atencion
farmaceutica':jt OR 'athletic therapy today':jt OR 'atmospheric
environment':jt OR 'audiological medicine':jt OR 'australasian
biotechnology':jt OR 'australia and new zealand health
policy':jt OR 'australian journal of forensic sciences':jt OR
'australian journal of medical science':jt OR 'australian
journal of pharmacy':jt OR 'australian journal of primary
health':jt OR 'australian prescriber':jt OR 'awic bulletin':jt
OR 'azerbaijan medical journal':jt OR 'bag - journal of basic
and applied genetics':jt OR 'bahrain medical bulletin':jt OR
'balkan journal of medical genetics':jt OR 'bangladesh journal
of dermatology, venereology and leprology':jt OR 'bangladesh
journal of obstetrics and gynecology':jt OR 'bangladesh renal
journal':jt OR 'basic and clinical pharmacology and toxicology,
supplement':jt OR 'behavioral and brain functions':jt OR
'behaviour change':jt OR 'behavioural and cognitive
psychotherapy':jt OR 'best practice and research in clinical
anaesthesiology':jt OR 'biocatalysis and biotransformation':jt
OR 'biochemia medica':jt OR 'biochemical engineering journal':jt
OR 'biochemistry, supplemental series a':jt OR 'biochemistry,
supplemental series b':jt OR 'biofilms':jt OR 'biogenic
amines':jt OR 'biological procedures online':jt OR 'biological
rhythm research':jt OR 'biologische medizin':jt OR 'biomarker
insights':jt OR 'biomarkers in medicine':jt OR 'biomedical
engineering':jt OR 'biomedical engineering - applications, basis
and communications':jt OR 'biomedical materials':jt AND [1980-
2009]/py

/* Combining all EMBASE-only journals */
30. 21 OR 22 OR 23 OR 24 OR 25 OR 26 OR 27 OR 28 OR 29

/* Restricting search results to EMBASE-only journals */
31. 20 AND 30
```

## II.4. Table with attribution of search strategies instructions to the fields of epidemiology

| Attribution of search strategies instructions to the kidney/urinary disease, to the epidemiology in general, to the merging or definition of limits | Number(s) of instructions in the search strategy |
|---|---|
| **Free word search strategy (FreeWoSS) for PubMed** | |
| Kidney/urinary diseases | 1-6, 8 |
| Epidemiology in general | 7, |
| Definition of limits/exclusion terms | 9-13 |
| Merging | 14-22 |
| **Subject headings search strategy (SuHeSS) for PubMed** | |
| Kidney/urinary diseases | 2-7 |
| Epidemiology in general | 8 |
| Definition of limits/exclusion terms | 1, 9-13 |
| Merging | 14-22 |
| **Search strategy for EMBASE** | |
| Kidney/urinary diseases | 1-4, 6 |
| Epidemiology in general | 5 |
| Definition of limits/exclusion terms | 7, 13-18, 21-29 |
| Merging | 8-12, 19-20, 30-31 |

# III. Methodology and interface of different steps of the systematic review

## III.1. Results of a trial search using both FreeWoSS and SuHeSS for the condition "Chronic kidney disease"

After formulating two search strategies we performed a trial search using both FreeWoSS and SuHeSS for 'Chronic kidney disease', and evaluated the first 1000 records obtained according to pre-defined criteria for potential usefulness in order to choose a single strategy for all our conditions of interest. We found that each strategy alone would exclude a rather substantial number of potentially useful literature data sources. Thus, while reviewing the first 1000 records obtained using SuHeSS only (SuHeSS NOT FreeWoSS) we found 3 potentially useful references (0.3% of all references in this set, 95% CI 0.07-0.95). Our review of the first 1000 records obtained using FreeWoSS only (FreeWoSS NOT SuHeSS) resulted in our finding 11 original articles and 11 reviews or editorials that could potentially be useful (2.2% of all references in this set, 95% CI 1.4-3.4). Considering the tens of thousands of records expected, it was possible to extrapolate that each search strategy alone would exclude at least 30-200 potentially useful records, and would thus prevent us from obtaining the maximum possible amount of information for global evidence of disease burden, especially for countries or populations with a paucity of published results on the epidemiology of kidney and urinary diseases. Due to this, we decided to apply both search strategies for all our conditions and to obtain a comprehensive set of bibliographic records.

## III.2. Criteria for the allocation of records to 'potentially useful' or 'not useful' during the step 3 of the systematic review

**The first estimate of selected literature is based on evaluation of article title and abstract.**

1. The abstract title should be *rejected* if it does not consider the incidence, prevalence, mortality, survival, complications (cardiovascular diseases, cancer, infections etc) of the following conditions:
    a. **Acute and chronic glomerulonephritis** (including acute or chronic renal failure, end-stage renal disease as sequelae);

   b. **Chronic pyelonephritis and tubulointerstitial nephritis** (including chronic renal failure, end-stage renal disease as sequelae);
   c. **Urolithiasis** (including acute or chronic renal failure, end-stage renal disease as sequelae);
   d. **Diabetic nephropathy** (but not diabetes itself or any other complications of diabetes);
   e. **Chronic kidney disease**
   f. **Benign prostatic hypertrophy.**
2. The abstract title should be *rejected* if it considers the following kidney diseases:
   a. Polycystic kidney disease and other hereditary nephropathies;
   b. Congenital genitourinary disorders;
   c. Tumors (malignant or benign) of the kidney, ureter or bladder;
   d. Hypertensive kidney disease;
   e. Kidney involvement in systemic vasculitis (lupus erythematosus, hemolytic-uremic syndrome, etc);
   f. Acute urinary tract infections.
3. The abstract title should be *rejected* if it considers modifications by lifestyle or medical intervention of the aforementioned conditions (including controlled randomized trials).
4. The abstract title should be *rejected* if it mentions the above topics in patients with very special conditions (such as if the abstract includes only patients with non-kidney transplants, recurrent glomerulopathies in kidney transplant patients, nephropathy in talassemia, etc).
5. The abstract title should be *rejected* if it considers only case studies or fewer than 50 patients.
6. The abstract title should be *rejected* if it considers experimental or in vitro studies.
7. The abstract title should be *rejected* if it considers acute renal failure related to exogenous substances (contrast media, drugs etc), sepsis, pancreonecrosis and other conditions not related to the list of conditions mentioned above.
8. The abstract title should be *rejected* if it considers only narrow topics in the field of kidney diseases, such as only anemia, only mineral disorders, etc.
9. The abstract title should be marked as *potentially useful* if it contains data on the topics mentioned above.
10. The abstract title should be marked as *potentially useful* if it presents data from registries of kidney disease, chronic renal failure, acute renal failure (not only due to the

aforementioned causes), diabetes (not only diabetic nephropathies), urological pathologies.

11. The abstract title should be marked as *potentially useful* if the reviewer has any doubts about the abstract's usefulness.

## III.3. Results of between-reviewer agreement assessment for classification of records at the 3 step of the systematic review

Table S1. Results of estimation the common set of 504 records by four reviewers at the step 3 of the systematic review

| | Reviewer 1 | Reviewer 2 | Reviewer 3 | Reviewer 4 |
|---|---|---|---|---|
| Sensitivity | 77.8 | 88.9 | 88.9 | 55.6 |
| Specificity | 98.9 | 94.5 | 97.8 | 94.5 |

# III.4. Interface for preliminary data extraction based from the abstract at the 5 step of the systematic review



**Abstract estimation form**

**Country of description:**

**Article language:**

**Number of reported persons/patients**

**Article type is:**

- ○ original article
- ○ review article with data from developed world
- ○ review article with data from developing world
- ○ review article without mentioning the region of description
- ○ review article without abstract (title suggests usefulness)
- ○ registry report
- ○ official report for health authorities
- ○ other

**Study population coverage:**

- ○ single center experience
- ○ community
- ○ city
- ○ province / state
- ○ sub-national
- ○ national
- ○ multi-national
- ○ not known

**Study sampling strategy:**

- ○ Convinient sampling (not random/systematic sampling) or not known
- ○ Any type of systematic sampling
- ○ Any type of random sampling
- ○ Census (registry report)

**Details on sampling population:**

- ☐ Healthy population
- ☐ Sampling of persons admitted to the primary care hospital / general practicions
- ☐ Sampling of persons admitted to the secondary/tertiary care hospital
- ☐ Sampling only patients with specific disease
- ☐ Sampling of selected specific population
- ☐ Sampling of only CKD or ESRD patients
- ☐ biopsy study
- ☐ other
- ☐ not known

**CKD stages / patients reported in the study:**

- ☐ CKD stage 1
- ☐ CKD stage 2
- ☐ CKD stage 3
- ☐ CKD stage 4
- ☐ CKD stage 5 (not on RRT)
- ☐ CKD with detalization other than stages 1-5
- ☐ CKD in total (without information on stages)
- ☐ hemodialysis patients
- ☐ peritoneal dialysis patients

- ☐ kidney transplant patients
- ☐ incident ESRD population
- ☐ not known

**Study period:**
start ___ end ___ ☐ not reported

**Studied population age:**
- ○ only children
- ○ only adults
- ○ both children and adults
- ○ not reported

Age range: min ___ max ___

**Studied population sex:**
- ○ only males
- ○ only females
- ○ both males and females
- ○ not reported

**Studied population urbanicity:**
- ○ only rural
- ○ only city
- ○ both city and rural
- ○ not reported

**Study design:**
- ○ case-control study
- ○ cross-sectional study
- ○ retrospective cohort study
- ○ prospective cohort study
- ○ randomized controlled trial
- ○ registry report
- ○ not reported

**Epidemiological parameters in abstract:**
- ☐ incidence ☐ potentially reported
- ☐ prevalence ☐ potentially reported
- ☐ outcome ☐ potentially reported
  - ☐ patient mortality
  - ☐ patient survival
  - ☐ ESRD rate
  - ☐ renal survival
  - ☐ cardiovascular events rate
  - ☐ other clinically important events (sepsis, etc)
  - ☐ remission
- ☐ prevalence or incidence of anemia
- ☐ prevalence or incidence of mineral disturbances
- ☐ prevalence or incidence of other metabolic abnormalities
- ☐ prevalence or incidence of diabetes complications other then nephropathy

**Nephrological (urological) abstract topics:**
- ☐ only CKD in total without specifying nosology
- ☐ CKD with kidney diseases nosology evaluation after initial screening
- ☐ chronic glomerulonephritis
- ☐ acute glomerulonephritis
- ☐ chronic pyelonephritis

- ☐ chronic tubulointersticial nephritis
- ☐ urolithiasis
- ☐ congenital GU malformations
- ☐ heredetary kidney diseases
- ☐ hypertensive kidney disease
- ☐ diabetic nephropathy in type 1 DM
- ☐ diabetic nephropathy in type 2 DM
- ☐ diabetic nephropathiy without DM type detalisation
- ☐ benign prostatic hypertrophy
- ☐ end-stage renal disease
- ☐ acute renal failure
- ☐ other

**Disease ascertainment was estimated based on:**

- ☐ Calculated Glomerular filtration rate
- ☐ Measured Glomerular filtration rate
- ☐ Serum creatinine ; SCr treshold μmol/l
- ☐ Microalbuminuria
- ☐ Albuminuria (or albumin/creatinine ratio)
- ☐ Proteinuria (or protein/creatinine ratio)
- ☐ Hematuria
- ☐ Other urinary abnormalities
- ☐ Imaging abnormalities (ultrasound, CT, etc)
- ☐ Patient interview
- ☐ Patient self-completed questionnaries
- ☐ Kidney biopsy
- ☐ Autopsy
- ☐ Prostate rectal examination
- ☐ Prostate ultrasound
- ☐ Prostate-specific antigen determination
- ☐ Urinary flow rate measurement
- ☐ not known

**Does study include follow-up:**

- ○ yes
- ○ no / not known

**Do risk factors for CKD in studied population are reported:**

- ○ yes
- ○ no / not known

**Subjective estimation of reviewer on abstract quality:**

- ○ 1 (lowest)
- ○ 2
- ○ 3
- ○ 4
- ○ 5 (highest)

**Comments on study quality:**

Submit abstract estimation

## III.5. Interface for selection of articles for full-text retrieval and further data extraction at the 6 step of the systematic review



# GBD Genitourinary Diseases Group

Welcome, Boris Bikbov
Log out | Change details
Your activities
Estimate abstracts
View your statistics
Submit new data source
Goto Admin panel
Menu
Main page
About GBD
About GBD GU Group
How can you participate?
Current activity
Help
Contact us
Credits

Sort abstracts: default (by country, then by population size)

All abstracts for "ASIA PACIFIC, HIGH INCOME" region, "only CKD in total without specifying nosology" condition - 32 abstract(s)

Prevalence of chronic kidney disease (CKD) in the Japanese general population predicted by the MDRD equation modified by a Japanese coefficient
Imai, E., Horio, M., Iseki, K., Yamagata, K., Watanabe, T., Hara, S., Ura, N., Kiyohara, Y., Hirakata, H., Moriyama, T., Ando, Y., Nitta, K., Inaguma, D., Narita, I., Iso, H., Wakai, K., Yasuda, Y., Tsukamoto, Y., Ito, S., Makino, H., Hishida, A., Matsuo, S. - Clin Exp Nephrol. 2007; 11 (2): 156-163 (show abstract)
Abstract was estimated by: monica cortinovis (id 10, level 40, birthday 19810831, email cortinovis@marionegri.it) from 105 (change estimation)
Country: Japan
Number of patients: **527594**
original article, coverage: national, Sampling of selected specific population (adult population aged over 20 years)
Sampling: Convinient sampling (not random/systematic sampling) or not known, study design: retrospective cohort study
CKD stage(s): CKD stage 3, CKD stage 4, CKD stage 5 (not on RRT)
Ascertainment based on: Calculated Glomerular filtration rate, Proteinuria (or protein/creatinine ratio)
Epidemiological param(s): prevalence
Study period: 2000-2004
Study population age: only adults (20-89), sex: both males and females, urbanicity: not reported
Study includes follow-up: no / not known, other risk factors are reported: yes
Subjective abstract estimation: 3
Your decision: - please select from drop-down menu -
- please select from drop-down menu -
include in full-text estimation
this data source is not useful as full-text

Estimated number of patients with chronic renal failure but not with end-stage renal disease in Japan: comparisons between two estimation methods
Wakai, K., Nakai, S., Shinzato, T., Kawamura, T., Tamakoshi, A., Aoki, R., Kojima, M., Lin, Y., Nakayama, T., Maeda, K., Ohno, Y. - J Epidemiol. 1998; 8 (5): 278-284 (show abstract)
Country: Japan
Number of patients: **296000**
registry report, coverage: national, Sampling of only CKD or ESRD patients
Sampling: Census (registry report) , study design: registry report
CKD stage(s): CKD in total (without information on stages)
Ascertainment based on: not known
Epidemiological param(s): incidence, prevalence, outcome, patient mortality
Study period: unknown
Study population age: not reported, sex: not reported, urbanicity: not reported
Study includes follow-up: no / not known, other risk factors are reported: no / not known
Subjective abstract estimation: 3
Your decision: - please select from drop-down menu -

## III.6. Interface for data extraction from full-text articles at the 7 step of the systematic review

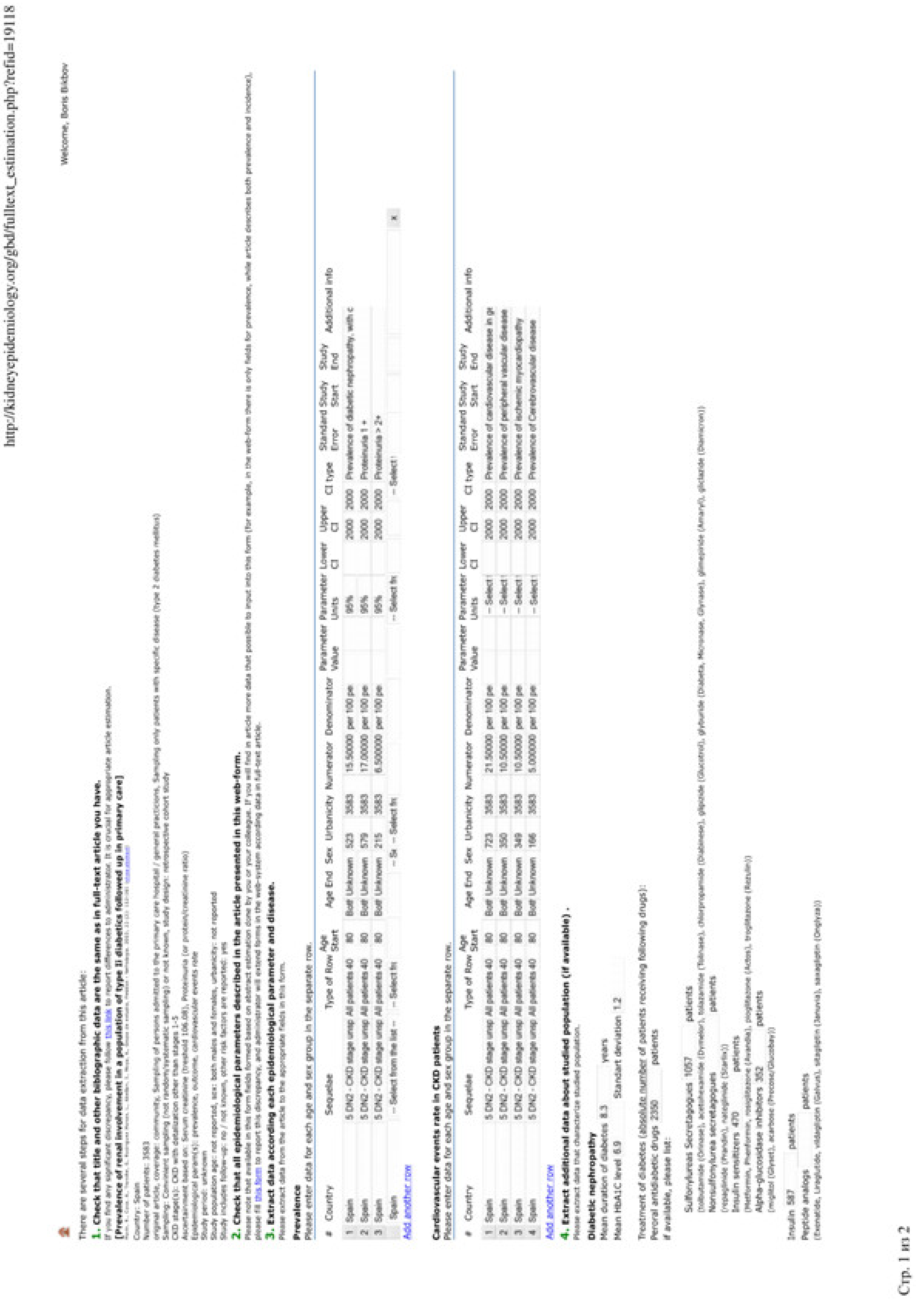

Initial changes were conformed by repetition of tests in patients with revealed abnormalities:
- [x] no
- [ ] yes, ones in ____ patients
- [ ] yes, two or more times in ____ patients
- [ ] yes, ones in all patients with initially revealed CKD patients
- [ ] yes, two or more times in all patients with initially revealed CKD patients

Risk factors of CKD in whole studied population
Age 64.0 (SD 10.0 ) years
Systolic BP 150.0 (SD 23.0 ) mm Hg
Diastolic BP 84.0 (SD 12.0 ) mm Hg
% with hypertension 69.0 %
BMI 29.2 (SD 4.0 )
% with BMI 25-30
% with BMI >30 38.0
% with increased W/H
Cholesterol 5.6 (SD 1.1 ) (mmol/l)
% with dyslipidemia
% of current smokers 17.0
% of former smokers 17.0
% of patients with CVD 21.5
% of poverty population
poverty treshhold USD

Does any renoprotective intervention was performed during follow-up period?
- [ ] no
- [ ] yes
- [ ] unknown

If yes, please fill:
- [ ] salt restriction
- [ ] weight correction
- [ ] other lifestyle modification
- [ ] ACE inhibitors
- [ ] Angiotensin II receptor antagonists
- [ ] diuretics
- [ ] Beta-blockers
- [ ] dihydropyridine Ca-channel blockers
- [ ] non-dihydropyridine Ca-channel blockers
- [ ] alfa-blockers

other

**5. Save the data you input to the web-form.**
Please note this button save data you enter and it will be stored in the web-system, but click on the "Save" button doesn't mean you submit your extraction to the final estimation. By saving the data, you can return to data editing whenever you wish.
Save

**6. Submit final extraction to the database.**
Please note that only after this final submission extracted by you data will be considered for final analysis. Before you click "Final submit" button administrator consider you are still editing data in this form.
Final submit

# IV. Methodology for comparing different PubMed search strategies

Sensitivity was calculated as the proportion of full-text articles used for data extraction in a given search for these articles in the PubMed ‘gold standard’ set, and thus measured the proportion of positives that were correctly identified. The higher the sensitivity, the more successful the strategy for capturing the articles with epidemiological information for data extraction.

Specificity was calculated as the proportion of records excluded from the full-text data extraction in a given search for the number of these records in the PubMed ‘gold standard’ set, and reflected the ability of the search strategy to correctly identify records that were not useful.

Precision was calculated as the proportion of records used for the full-text data extraction out of the total number of records obtained using a search strategy. In a diagnostic test, the term ‘precision’ refers to a positive predictive value. Number needed to retrieve could also be calculated as 1 divided by precision.

Accuracy was calculated as the proportion of all records correctly obtained using a search strategy with regard to all the records obtained by the ‘gold standard’ PubMed set.

Table S2. Contingency table comparing PubMed filter to the reference ‘gold standard’ set

| | | ‘Gold standard’ set | |
|---|---|---|---|
| | | full-text article with data extracted | articles not useful for data extraction |
| Single search strategy | identified by search strategy | a | b |
| | not identified by search strategy | c | d |

Referring to the table S2 performance indicators for a search strategy were calculated as follows:

Sensitivity = $100 * a / ( a + c )$

Specificity = $100 * d / ( b + d )$

Precision = $100 * a / ( a + b )$

Accuracy = $100 * ( a + d ) / ( a + b + c + d )$

# V. Guidelines for Accurate and Transparent Health Estimates Reporting (GATHER) recommendations checklist

Table S3. GATHER checklist of information that should be included in reports of global health estimates

| Item # | Checklist item | Reference |
|---|---|---|
| **Objectives and funding** | | |
| **1** | Define the indicator(s), populations (including age, sex, and geographic entities), and time period(s) for which estimates were made. | Manuscript (Methods) |
| **2** | List the funding sources for the work. | Manuscript (Funding) |
| **Data Inputs** | | |
| *For all data inputs from multiple sources that are synthesized as part of the study:* | | |
| **3** | Describe how the data were identified and how the data were accessed. | Manuscript (Methods), and Supplement (sections I and II) |
| **4** | Specify the inclusion and exclusion criteria. Identify all ad-hoc exclusions. | Manuscript (Methods), and Supplement (sections I and II) |
| **5** | Provide information on all included data sources and their main characteristics. For each data source used, report reference information or contact name/institution, population represented, data collection method, year(s) of data collection, sex and age range, diagnostic criteria or measurement method, and sample size, as relevant. | Online data citation tool http://ghdx.healthdata.org/gbd-2016/data-input-sources |
| **6** | Identify and describe any categories of input data that have potentially important biases (e.g., based on characteristics listed in item 5). | Manuscript (Methods), and Supplement (section I) |
| *For data inputs that contribute to the analysis but were not synthesized as part of the study:* | | |
| **7** | Describe and give sources for any other data inputs. | Not presented |
| *For all data inputs:* | | |
| **8** | Provide all data inputs in a file format from which data can be efficiently extracted (e.g., a spreadsheet rather than a PDF), including all relevant meta-data listed in item 5. For any data inputs that cannot be shared because of ethical or legal reasons, such as third-party ownership, provide a contact name or the name of the institution that retains the right to the data. | Search strategies are presented in Supplement (section I), Finally selected data sources are available via data citation tool http://ghdx.healthdata.org/gbd-2016/data-input-sources, with the limitation to the systematic review period |
| **Data analysis** | | |
| **9** | Provide a conceptual overview of the data analysis method. A diagram may be helpful. | Manuscript (Methods)and Supplement (section III) |
| **10** | Provide a detailed description of all steps of the analysis, including mathematical formulae. This description should cover, as relevant, data | Manuscript (Methods) and Supplement (sections I and II) |

| | | |
|---|---|---|
| | cleaning, data pre-processing, data adjustments and weighting of data sources, and mathematical or statistical model(s). | |
| **11** | Describe how candidate models were evaluated and how the final model(s) were selected. | Manuscript (Methods) and Supplement (section II) |
| **12** | Provide the results of an evaluation of model performance, if done, as well as the results of any relevant sensitivity analysis. | Manuscript (Methods) and Supplement (section II) |
| **13** | Describe methods for calculating uncertainty of the estimates. State which sources of uncertainty were, and were not, accounted for in the uncertainty analysis. | Manuscript (Methods) and Supplement (section III) |
| **14** | State how analytic or statistical source code used to generate estimates can be accessed. | Code could be provided on request |
| **Results and Discussion** | | |
| **15** | Provide published estimates in a file format from which data can be efficiently extracted. | Manuscript, Supplement |
| **16** | Report a quantitative measure of the uncertainty of the estimates (e.g. uncertainty intervals). | Manuscript, Supplement |
| **17** | Interpret results in light of existing evidence. If updating a previous set of estimates, describe the reasons for changes in estimates. | Manuscript (Methods and Discussion) |
| **18** | Discuss limitations of the estimates. Include a discussion of any modelling assumptions or data limitations that affect interpretation of the estimates. | Manuscript (Methods) |
